# 6G Enabled Smart Infrastructure for Sustainable Society: Opportunities, Challenges, and Research Roadmap

**DOI:** 10.3390/s21051709

**Published:** 2021-03-02

**Authors:** Agbotiname Lucky Imoize, Oluwadara Adedeji, Nistha Tandiya, Sachin Shetty

**Affiliations:** 1 Department of Electrical Engineering and Information Technology, Institute of Digital Communication, Ruhr University, 44801 Bochum, Germany; 2 Department of Electrical and Electronics Engineering, Faculty of Engineering, University of Lagos, Akoka, Lagos 100213, Nigeria; 3 Department of Electrical and Electronic Engineering, Faculty of Technology, University of Ibadan, Ibadan 200284, Nigeria; padedeji314@stu.ui.edu.ng; 4 Bradley Department of Electrical and Computer Engineering, Virginia Polytechnic Institute and State University, Blacksburg, VA 24061, USA; nistha@vt.edu; 5 Virginia Modelling Analysis and Simulation Center, Department of Computational Modelling and Simulation Engineering, Old Dominion University, Norfolk, VA 23529, USA; sshetty@odu.edu

**Keywords:** 6G wireless communication, 6G vision, 6G requirements, 6G enabling technologies, 6G challenges, 6G applications, 6G use cases, 6G infrastructure, 6G sustainability, 6G business model

## Abstract

The 5G wireless communication network is currently faced with the challenge of limited data speed exacerbated by the proliferation of billions of data-intensive applications. To address this problem, researchers are developing cutting-edge technologies for the envisioned 6G wireless communication standards to satisfy the escalating wireless services demands. Though some of the candidate technologies in the 5G standards will apply to 6G wireless networks, key disruptive technologies that will guarantee the desired quality of physical experience to achieve ubiquitous wireless connectivity are expected in 6G. This article first provides a foundational background on the evolution of different wireless communication standards to have a proper insight into the vision and requirements of 6G. Second, we provide a panoramic view of the enabling technologies proposed to facilitate 6G and introduce emerging 6G applications such as multi-sensory–extended reality, digital replica, and more. Next, the technology-driven challenges, social, psychological, health and commercialization issues posed to actualizing 6G, and the probable solutions to tackle these challenges are discussed extensively. Additionally, we present new use cases of the 6G technology in agriculture, education, media and entertainment, logistics and transportation, and tourism. Furthermore, we discuss the multi-faceted communication capabilities of 6G that will contribute significantly to global sustainability and how 6G will bring about a dramatic change in the business arena. Finally, we highlight the research trends, open research issues, and key take-away lessons for future research exploration in 6G wireless communication.

## 1. Introduction

The commercialization of 5G commenced in 2019, and further adoption is expected in 2021 and beyond. There have been tangential research interest on future 6G wireless networks [1]. The COVID-19 pandemic forced more businesses online, birthing a “New Normal” with a borderless workplace. Ericsson has forecasted that there will be faster commercialization of 5G as more people embrace this shift to a borderless workplace. The resulting surge in Internet usage beams light on the need for better connectivity to meet the growing demand for more stringent network requirements. This is required to facilitate emerging technologies such as extended reality [2], haptics [3], connected autonomous systems [4], telemedicine, Industrial Internet of Things (IIoT) [5], which are sensitive to latency and require ultra-fast data speed. For example, ultra-low latency and ultra-fast data speed reduce the collision rates and improve the safety of the autonomous vehicle. These applications are necessary to facilitate autonomous and smart life, multisensory virtual experience, intelligent cities, smart agriculture, and more. Unfortunately, the promising 5G networks cannot meet these growing demands [6,7]. Thus, there is an imperative need for the development of 6G communication networks. 6G wireless networks are also proposed to ameliorate social needs, thereby facilitating the actualization of the Sustainable Development Goals (SDGs) [8]. The proposed network requirements of 6G can be summarized as (1) Ultra-fast data rates as high as 1Tbps (2) Ultra-low latency of less than 1ms (3) Increased mobility and coverage (4) Flexible, and efficient connection of trillion level objects [9] (5) Peak spectral efficiency of 60 b/s/Hz (6) Very high system reliability (7) Improved network security.

6G is expected to have data-rates in the order of terabits per second and a latency of less than 1ms. It is expected to drive the Internet of Everything, with 10^7^ connections per km^2^ [10]. To achieve this, 6G will leverage on subterahertz and Terahertz spectrum (300 GHz to 10 THz) [11,12], which provides a higher frequency spectrum as against the millimeter wave spectrum (30–300 GHz) adopted in 5G [13]. Exploring a higher frequency spectrum is necessary because the sub-6GHz range is already crowded. Apart from giving room for more spectrum, the Terahertz spectrum gives rise to higher data rates desirable in 6G networks. However, transmitting at a higher frequency spectrum is prone to high path loss, making the distance for transmission limited. This and other challenges with THz transmissions, such as hardware constraints, are treated in this paper. Additionally, Optical wireless technologies [14] such as Visible Light Communication (VLC) [15] and Free Space Optical communication [16] are discussed extensively.

Additionally, technologies such as Reconfigurable Intelligent Surfaces (RIS) [17,18,19], cell-free massive MIMO [20], Artificial Intelligence (AI), which are expected to drive the actualization of 6G, are broached. We consider RIS, which will be deployed on doors, windows of buildings to reflect received signal without interference. Furthermore, we explore why the RIS technology is a preferable candidate to the existing relays. The massive MIMO technology is introduced in 5G with a more dense network of access points (APs) [21]. This is further developed in 6G to include a network with no cells (cell-free) [22]. The benefits are tremendous as it improves spectral efficiency in communication networks. However, there are challenges with obtaining channel information and concerns about health risks associated with such a dense network of APs. There is limited literature to address these concerns; thus, the need for this review. We also believe Pervasive AI is critical in actualizing 6G. Artificial Neural Networks [23,24], Deep Neural Networks [25] have been proposed to enable intelligent networks.

Despite the auspicious view of this technology, challenges with complex data and more have been highlighted. These challenges have been delineated in this article. Other enabling technologies such as Quantum Communication [26], Ambient Backscatter Communication Systems (ABCS) [27], Blockchain [28], UAVs [29], and more have also been proposed and treated extensively in this paper. Future generation networks are desired to have high speed and low latency and secured communication. Quantum communication has been proposed to enable security and facilitate faster processing power for future wireless networks [30]. However, there are doubts if research in quantum communication will be ripe enough to facilitate the 6G communication systems [31]. However, this technology will see more light in the beyond 6G, towards the 7G era. Blockchain is another technology proposed to facilitate security, and we have examined this with the hope that it would provide the desired privacy and integrity in future wireless networks. Blockchain technology has been introduced in 5G [32], and further research on the challenges treated in Section 5 of this paper would facilitate the security of future wireless networks.

Energy efficiency is another interesting topic for future wireless networks. It is desirable to have hardware that is compatible with the energy requirements of 6G. Ambient Backscatter Communication Systems (ABCS), an Energy Harvesting (EH) technique, is proposed to enable wireless charging. This gives room for longer battery life, which has been proposed as a requirement for future wireless networks [33]. With the ABCS, devices have an alternative source of power from wireless communication. This is consequently extending the battery life of devices. Simultaneous Wireless and Information Power Transfer (SWIPT) [34], if enabled by 6G, will resolve energy requirements issues at the mobile unit [35]. We believe this will enable haptics [36], the Internet of Bio-Nano Things, and other applications with very restrictive energy requirements.

Seamless and fast ubiquitous communication is also desirable in 6G. UAVs and CubeSats, which form the Internet of Space things [37], will facilitate this. We have considered the benefits of CubeSats compared to the conventional satellites, for example, their low cost is desirable [38]. Challenges with UAVs such as network handover management and how advanced technologies such as AI interface can solve these challenges are treated in detail in this paper.

This paper provides researchers with a guide to what 6G will be. It proposes a panoramic vision of 6G, the enabling technologies, and the associated challenges. This makes the paper to be robust, compensating for the individual lapses in the existing literature. Additionally, we provide an extensive analysis of the social, psychological, health, and commercialization challenges associated with 6G, which are missing in earlier reports to the authors’ best knowledge. The article also introduces new use cases of 6G in education, media and entertainment, tourism and transport, and logistics, which have not been given adequate treatment in the existing literature. Finally, we discuss the multi-faceted communication capabilities of 6G that will contribute immensely to global sustainability. We discussed how 6G would bring about drastic changes in the business domain to orchestrate economic stability.

**Figure 1:**
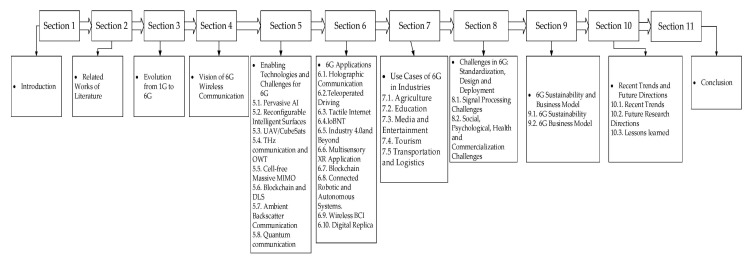
This is a figure showing the layout of the paper.

Figure 1 presents the layout of the paper. In summary, the key discussions in the paper are as follows:

1. The vision of 6G wireless networks, considering the essential requirements and comparing them with the existing generations of wireless communication networks.
2. A comprehensive review of the 6G enabling technologies such as Pervasive AI, Reconfigurable Intelligent Surfaces, Ambient Backscatter Communication, and more.
3. The technical challenges associated with the enabling technologies and the non-technical challenges such as social, psychological, commercialization, and health concerns towards the actualization of 6G wireless communication.
4. New applications and use cases in agriculture, education, media and entertainment, tourism, transport, and logistics to be enabled by 6G.
5. Harnessing the potentials of 6G to meet the UN sustainability goals and business model tailored towards attaining global economic stability.
6. The recent trends and future research directions for 6G wireless communication. We identified some open research issues and summarized the proposed research focus.
7. Finally, the lessons learned from the survey are delineated, and the conclusion to the paper is presented.

The rest of this paper is organized as follows: Section 2 presents the related works of literature. Section 3 presents the evolution of wireless networks, from 1G to 6G. The technologies facilitating each generation are introduced, and a comparative analysis of 5G, beyond 5G (B5G) and 6G, is presented. The proposed 6G vision is delineated in Section 4. The paper goes further to treat the 6G enabling technologies and the associated challenges in Section 5. Applications of 6G such as Holographic communication, Multisensory (XR), Tactile Internet, and more are presented in Section 6. New 6G use cases in agriculture, education, media and entertainment, logistics and transportation, and tourism are introduced in Section Section 7. In addition to the challenges of the enabling technologies, this paper further delineates the challenges associated with commercialization, social, health, and psychological issues in Section Section 8. 6G sustainability and business model is presented in Section 9. Future research areas and trends are broached, and the lessons learned are summarized in Section 10. Finally, the conclusion to the paper is given in Section 11.

## 2. Related Works and Contributions

There are a few related works of literature that proposed the vision, requirements, enabling technologies, and design of 6G wireless networks [33,39,40,41,42]. In particular, David et al. [33] opined that 6G would enable wireless charging and high data rates. The authors also identified the need for socio-ethics in the 6G design. Nayak and Patgiri [39] proposed 6G to change the perceptual experience in lifestyle, business, and society. The study also presents some technology-driven challenges in 6G wireless networks and the probable solutions. In [40], the authors examined critical features such as security, secrecy, and privacy to make 6G truly human-centric. Tariq et al. [41] present an extension to the existing vision of 5G and show speculatively how the 5G vision and technologies can be enhanced to drive the anticipated 6G. Yang et al. [42] proposed integrating machine learning and big data to facilitate intelligent transmission. The application of Big data and AI has also been considered in the scholarly works of literature [24,43,44]. Other enabling technologies such as Intelligent Reflecting Surfaces [17], Blockchain [28], Terahertz communication [11], and more, proposed for 6G communication, have also been surveyed. The vision and requirements proposed by different researchers are discussed extensively in Section 4 of this paper. Additionally, the future projections of wireless communication presented in existing reports are highlighted.

This paper extensively considers existing research, clearly outlining the enabling technologies and the associated challenges, applications, and new applications such as the IoBNT and Digital Replica, which are not given adequate treatment in many related papers. Use cases in agriculture, education, media and entertainment, and more are discussed extensively. Apart from the technical challenges associated with the enabling technologies, this paper examines the social, psychological, and health concerns that could pose a challenge to 6G adoption. This paper also explores the recent research breakthroughs in 5G and the limitations of 5G, which make 6G a highly prospective candidate. A comparative review of some of the proposed enabling technologies, open research issues and lessons learned, and proposed future research directions are also discussed extensively.

In summary, Table 1 examines the limitations of some of the existing surveys and our contributions in this paper to fill the knowledge gap. We hope this paper gives the reader a panoramic view of what 6G will be, clearly outlining the possible challenges associated with the 6G-enabling technologies, applications, use cases, and more. Finally, this paper provides a future outlook of what needs to be done to facilitate this desirable generation of wireless communication towards achieving the United Nations Sustainable Development Goals.

## 3. The Evolution from 1G to 6G

The analog wireless cellular network, which formed the first generation of wireless communication, was in use in the 1980s. This facilitated voice calls between mobile users. The Advanced Mobile Phone System (AMPS), International Mobile Telecommunications Standard (IMTS), and Point to Call formed the basis for the 1G. Additionally, some European countries adopted the Nordic Mobile Radio System (NMR). This resulted in compatibility challenges. The first-generation network was monopolized and was not affordable for many. 2G brought about high-quality, secure mobile voice and basic data services such as fax and text messaging. GSM was at the core of the 2G. It was regarded as the “Groupe Speciale Mobile”—a group of technical personnel set up by the Postal and Telecommunication Administration (CEPT) Conference to develop digital mobile communication technology [50]. This was developed as the wireless counterpart of the land-lined Integrated Services Digital Network (ISDN) system. The acronym was later changed to refer to “Global Systems for Mobile Communication.” The GSM standard was deployed in 1991, using the 900 MHz band [51].

The GSM architecture comprises the Mobile Station, Network and Switching Subsystem, and the Base Station Subsystem (BSS), also known as the radio network. Additionally, included is an intelligent network subsystem that enables intelligent functionality such as prepaid services and short message services (SMS). GSM utilized Frequency Division Multiple Access (FDMA) and Time Division Multiple Access (TDMA) for simultaneous communication between the subscriber and the base station [52]. The former allowed communication using multiple frequencies, while the latter enabled communication through multiplexing by time slots. General Packet Radio Service (GPRS) was developed to facilitate features such as always-on, higher capacity, internet-based content, packet-based data services, enabling services such as color internet service, email on the move, and visual communications, multimedia messages, and location-based services [53].

In the early twenty-first century, 3G was developed as an upgrade to the features in 2G. It permitted faster data rates in the range of 300 kbps–30 Mbps and services such as video conferencing, remote supervision systems, and enabled information services. Technologies such as Wideband Code Division Multiple Access (WCDMA), Universal Mobile Telephone Service (UMTS) were key to achieving the 3G. The 3rd Generation Partnership Programme (3GPP) was formed in 1998 to oversee UMTS implementation and other enabling technology for 3G. 3GPP2 was also formed in the United States to develop global specifications for 3G systems. Critical concepts for evolution toward beyond 3G networks are presented in [54].

Long-Term Evolution (LTE) was deployed in 2009. With the proliferation of smartphones and tablets, online gaming, and other services, 4G has been a significant success, enabling these services with its data speed of 100 Mps–1000 Mbps. Although the first release of LTE was in 2005 by 3GPP in release 6, the full development was only achieved in release 8 in 2008. Further details on the technical solutions for the 3G long-term evolution are reported in [55]. The LTE is often regarded as the 4G; however, the LTE-Advanced features such as increased peak data rate, spectral efficiency, simultaneous active subscribers, and improved cell-edge performance make it the true 4G. Key performance indicators for 4G LTE are given by [56,57], and radial basis function neural network pathloss prediction model in LTE network was reported in [58]. A higher data rate was achieved and lower latency in 20 ms–100 ms, which was lower than that obtained in 3G. 4G facilitated video streaming, online gaming, and more. The need for a higher data rate and lower latency gave room for interests in 5G.

5G commercialization started in 2019, and it opens up new use cases in the Internet of Things (IoT), immersive gaming, virtual reality, and more. 6G is expected to have a higher data rate in the range 100 Gbps–1 Tbps and latency lower than 1ms. This opens up applications in holographic communication, tactile internet, extended reality, and more. Table 2 summarizes the technology, data rates, and supporting applications from 1G to 6G. The change in latency from 1G to 6G is also shown. Furthermore, a comparative analysis of 5G, Beyond 5G (B5G), and 6G is presented in Table 3.

## 4. Vision of 6G Wireless Communication

There have been different descriptions of what the 6G network should be by researchers [40,45,79,80,81]. Furthermore, the authors in [82] defined it as a technology that will make human society a “Ubiquitous Intelligent Mobile Society.” In [41], the authors envisioned 6G to facilitate super smart cities with pervasive autonomous systems. It is expected that 6G will be supported by existing 5G infrastructure such as Software-Defined Networking [83], Network Function Virtualization (NFV) [84], and Network Slicing (NS) [85] together with new infrastructure. In order to give a future assessment of how well 6G has accomplished the required cases, this paper examines various visions and requirements projected by different researchers [42,43,44]. It presents a blend of what 6G will be. Just as we envisage, some researchers also believe there will be a pervasive application of AI to make 6G a reality [20,70]. There is also research on optical wireless communication to enable indoor and outdoor communication at high-data rates [86]. Simultaneous Wireless and Information Power Transfer (SWIPT) [35], which is an Energy Harvesting (EH) technique, has been proposed to improve the battery life of UEs [40,41].

6G is expected to support smart cities, the Internet of Everything (IoE) [39], tactile devices [9], and more. The requirements are high reliability [7], the high data rate in the order of 1 Tb/s, ultra-low latency of less than 1ms, high energy and spectral efficiency [87], security and privacy [88], and ubiquitous connectivity that connects everyone, including people in rural areas [89,90]. The Ultra-Reliable Low Latency Communication (URLLC) required in 6G networks is also under more stringent conditions than that obtainable in 5G, with delay jitters, context awareness, and UAV/Satellite compatibility being considered. Sustainability is also desirable in future wireless networks, and there is a need for Green Networking architecture that will be environmentally friendly [91].

**Figure 2:**
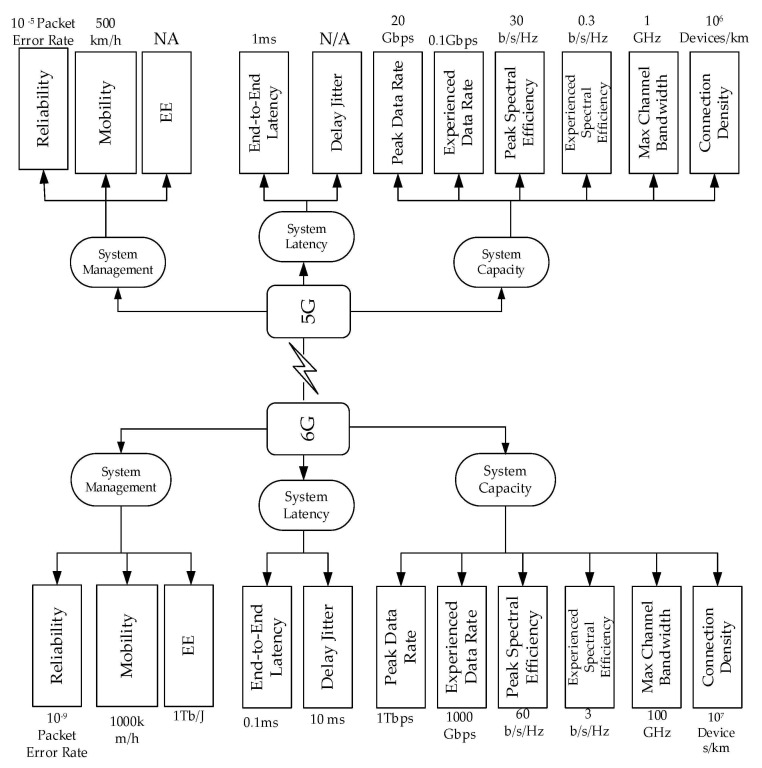
Comparative Analysis of 5G and 6G Key Performance Indicators.

Table 4 presents the vision and requirements projected by different researchers. This gives a sneak peek at the ongoing discussion on 6G networks. The focus and future projections are also highlighted. Services and Key Performance Indicators [92,93] are given, as shown in Table 5. Additionally, applications such as immersive extended reality, Brain–Computer Interface (BCI), Connected Robotic and Autonomous System (CRAS), and more, proposed to be supported by 6G, are presented in Table 6. Figure 2 shows a brief overview that compares the Key Performance Indicators in 5G and those expected in 6G.

**Figure 3:**
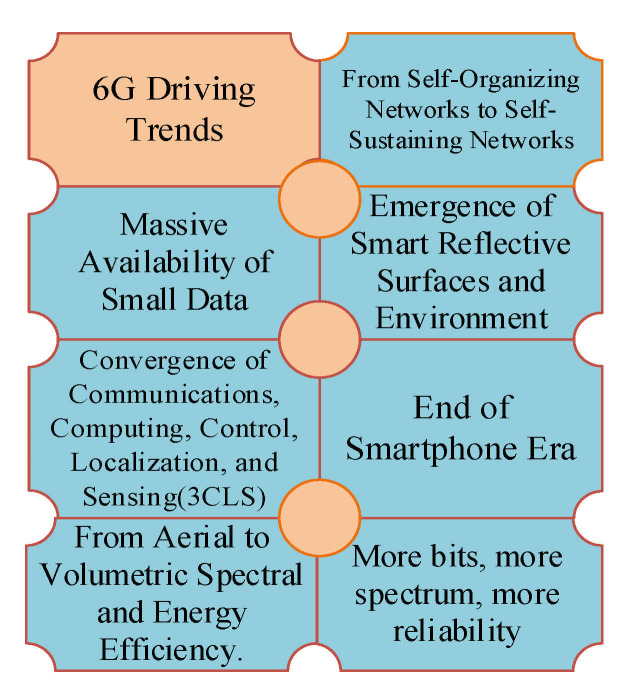
6G Driving Trends.

Furthermore, driving trends towards 6G are introduced in Table 6, and a pictorial guide of the driving trends towards 6G is shown in Figure 3. Since the flag-off of the 6G research project in Finland in May 2018 [94], many countries have been interested in 6G research. 6G research, though still in its fledgling state, has recorded positive results towards the achievement of this vision. Table 7 gives the initiatives in selected countries or research centers that carry out leading research in 6G and its progress.

**Table 4:** A summary of the vision and requirements of 6G wireless networks.

Ref.	Vision	Requirements	Focus	Future Projections
[47]	To support future IoE smart cities. It also provides a fully immersive experience of XR and Convergence of Communications, Computing, Control, Localization, and Sensing(3CLS).	A blend of URLLC and eMBB with perceptual factors from the user. Introduces a new concept QoPE. Multi-purpose 3CLS and Energy Services.	Human-centric services based on QoPE, Energy Services, and Mobile Broadband Reliable Low Latency Communications.	Quantum computing and Communications.
[40]	Enhanced conventional mobile communication, accurate indoor positioning, support new communication devices such as wearable devices, integrated and implantable sensors, quality network on aircraft, worldwide connectivity.	eMBB-Plus, SURLLC (URLLC+mMTC), massive Vehicle-to-Everything to support Robotic communication.	Security, Privacy, Energy Efficiency, Intelligent Networks, affordability and Customization, Trade-offs between key features and potential solutions.	The dependency on basic sciences, social and need for research on psychological factors could hamper commercialization.
[9]	Data-driven society enabled by almost instantaneous unlimited wireless connectivity. Supports fine telemedicine, intelligence disaster prediction, and surreal VR.	Undetectable or non-existent latency, Multi-band ultra-fast speed transmission, flexible integrated network, multi-mode multi-joint transmission, and Intelligent transmission.	Potential techniques for 6G networks and challenges associated with 6G communication.	Power supply, Network, and Hardware Design Issues.
[41]	Supports super smart cities, IoT, driverless cars, and pervasive empowerment by AI. Radar technologies will be integrated with mobile communication. Additionally, a possibility for wireless power transfer, RF energy harvesting, optical wireless communication and LiFi.	Increase in data rate from 1 Gbps in 5G to at least 10 Gbps per user, URLLC, better spectral and energy efficiency than 5G.	Use Cases, Challenges, and Key Enabling Technologies.	The shift from the electronic era in 5G to the optical and photonics era.
[6]	An ICT infrastructure enables end-users to perceive and be surrounded by a “huge artificial brain” offering virtually zero latency, unlimited storage, and immense condition capabilities. Vision for battery-free communication.	Exceedingly high reliability (1-10-9), extremely low latency (0.1–1 ms), and delay jitter. They are targeting communication efficiency on the order of 1 pJ/b.	Enablers of the 6G, distributed security mechanisms, Pervasive AI, and holistic management of Communication, Computation, Caching and Control (C4) resources.	Use of Blockchain, Pervasive application of AI, subterahertz, Visible Light Communication, and Optical Beamforming.
[48]	Supports smart wearables, implants, autonomous vehicles, computing reality devices, sensing, 3D Mapping.	The convergence of network densification, high throughput, high reliability, low power consumption, and capability for handling massive volumes of data and very high data rate connectivity per device. Ultra-long range communication with a latency of less than 1ms, low backhaul and access network congestion, AI, satellite integration, enhanced security with blockchain.	Vision, Requirements, Applications, Challenges, and Research Directions.	FSO communication, THz communication, Spectrum and Interference management, Autonomous Wireless systems, resource management.
[95]	Supports Mobile augmented reality, VR, Holographic teleconferencing, tactile internet of things, industrial IoT, intelligent driving.	Mobile broadband bandwidth and low latency (mBBLL), massive broadband bandwidth machine type (mBBMT), massive low latency machine type (mLLMT), 6G-Lite, security, Intelligence, energy, and spectral efficiency.	Evolution, 6G Enabling Technologies, Challenges.	Blockchain, Quantum computing, multidisciplinary affiliations with the physical and social sciences, intelligent 6G.
[45]	Seamless, ubiquitous connection, Teleoperation, cooperative and autonomous driving.	eMBB-Plus, Big communication, secure ultra-reliable low latency communication, 3D integrated communication, holographic, tactile, and human-bond communication.		FSO communication, THz communication, Spectrum and Interference management, Autonomous Wireless systems, resource management.
[81]	A large-dimensional and autonomous network architecture that integrates space, air, ground, and underwater. To provide a full sensory experience that requires a high volume of data, extremely high throughput, and very low latency. It also supports super-high definition and extremely high-definition (EHD) videos, Internet of NanoThings, and Internet of Bodies.	A peak data rate of at least 1 Tb/s, a user experience data rate of 1 Gb/s, and over-the-air latency of 10–100 μs and high mobility of at least 1000 km/h, ten times the connectivity of 5G and energy efficiency of 10–100 times and spectrum efficiency of 5–10 times those of 5G.	Vision, requirements, architecture, and key technologies.	Identified the following promising techniques: THz communications, SM-MIMO, LIS and HBF, OAM multiplexing, laser communications and VLC, quantum communications and computing, blockchain-based spectrum sharing, and Molecular communications and the Internet of NanoThings.
[80]	Intelligent Connectivity with pervasive AI, Deep connectivity(tactile internet, deep data mining, telepathy), Holographic connectivity(holographic communication, high fidelity, AR/VR), Ubiquitous connectivity(Space-Air-Ground-Sea network integration).	Massive connectivity, reliability, real-time and throughput requirements, reduced spectral efficiency, pervasive sensors.	Vision, requirement, challenges(technical and non-technical), and potential key technologies.	Future trends were not explicitly stated, but the paper discussed the key technologies required for 6G.
[33]	Wireless charging, Optical free-space communication, outdoor wireless communication.	Ultra-long battery lifetime, lesser focus on high bit rates.	Battery life, Regulations, and rules for frequency assignment, ethics surrounding the adoption of 6G.	Inclusion of human sensory information and emotion in future applications; also, sociotechnical designs to assess legal, psychological, and economic requirements.

This table gives a sneak peek into the discussion on the 6G vision and requirements. The table also serves as a guide to the focus and future projections by researchers. Technologies such as pervasive AI, blockchain are proposed by various researchers. Additionally, applications such as wireless charging and wearables are proposed. This overview is important as it helps keep track of what research thinks 6G would be, compare and contrast as the research progresses.

**Table 5:** Services, Key Performance Indicators (KPIs), and Applications of 6G.

Service	KPI	Applications
HCS	QoPE is capturing raw wireless metrics as well as human and physical factors.	Emphatic communication. BCI. Affective communication. Haptics.
MPS	Energy. Computing latency. Latency and reliability communication Control stability. Localization accuracy. Sensing and mapping accuracy.	Environmental mapping and imaging. Telemedicine. Connected Robotics and Autonomous System (CRAS). Some special cases of extended reality (XR) services.
MBRLLC	Energy efficiency. Stringent rate-reliability latency requirements. Rate-reliability latency in mobile environments.	Legacy eMBB and URLLC. XR/VR/ AR. Autonomous vehicular systems. Autonomous drones.
mURLLC	Scalable URLLC. Massive reliability. Ultra-high reliability. Massive connectivity.	Blockchain and DLT. Autonomous robotics. Massive sensing. Classical Internet of things. User tracking.

This table shows in detail the 6G services introduced in Table 3. Key Performance Indicators (KPIs) and enabling applications are also highlighted.

**Table 6:** A summary of the driving trends towards 6G wireless networks.

Driving Trends	Description
The convergence of Communications, Computing, Control, Localization, and Sensing (3CLS).	Provides computing, control, localization, and sensing in addition to Wireless Communication that previous generations provided. Supports applications such as XR, CRAS, DLS.
The emergence of Smart Reflective Surfaces and Environments.	Driven by smart reflective surfaces that serve as walls, roads, doors, and entire buildings, help maintain a line of sight and obtain a quality signal with minimal loss.
Massive Availability of Small Data.	The shift from centralized big data to massive distributed small data.
More bits, More spectrum, and More Reliability.	Exploring higher frequency spectrum (THz), which is proposed to facilitate the actualization of 1 Tb/s.
From Self-Organizing Networks to Self-Sustaining Networks.	AI is proposed to facilitate intelligent wireless networks that are self-sustaining.
Ubiquitous connectivity that encompasses air, ground, and undersea.	6G is envisioned to integrate space-air-ground-sea mode to facilitate wireless communication in flying vehicles, XR, BCI, and more.
The emergence of Haptics and the End of Smartphone era.	The pervasive use of wearables and implants, supported by BCI and XR.

This table highlights driving trends towards 6G. For example, the convergence of Communications, Computing, Control, Localization and Sensing (3CLS) or Communication, Computation, Caching and Control (C4) is a crucial driving trend towards 6G. Additionally, the emerging Smart Reflective Surfaces are expected to facilitate 6G. Other trends such as the emergence of haptics are also highlighted.

## 5. Enabling Technologies and Challenges for 6G

**Figure 4:**
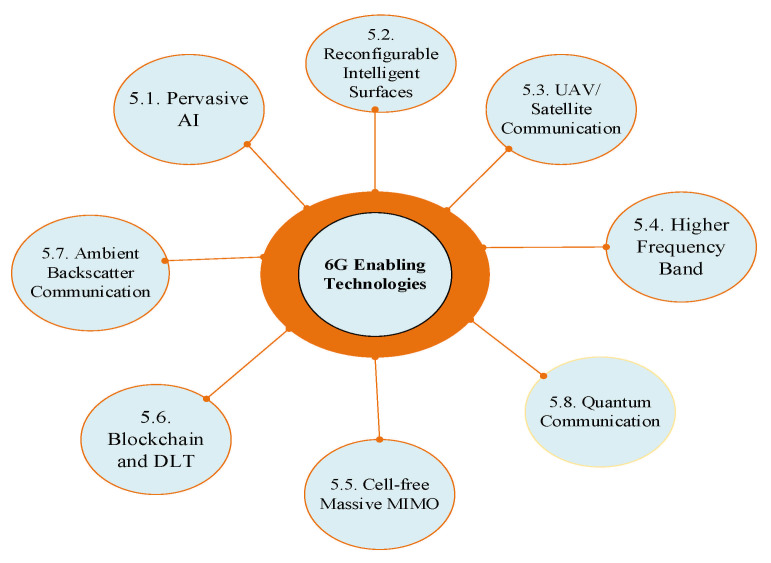
6G Enabling Technologies.

In this section, we present a robust discussion of the enabling technologies of the 6G communication system. We envision that Artificial Intelligence, which was introduced in 5G, will be further explored and central in achieving an intelligent 6G network. Reconfigurable Intelligent Surfaces will be deployed on doors, windows, buildings, and these reflect signals and help in places where maintaining Line of Sight (LoS) is tricky. The advantages of this over conventional relay systems are discussed. Cell-free Massive MIMO, TeraHertz, and Optical Wireless Technology, and more are also treated in this section. Quantum communication has been proposed, although research is in its inchoate state. This will improve the computing efficiency and security of future wireless networks. Unmanned Aerial Vehicles (UAV) and Cubesats are proposed to facilitate space communication. Some literature presents this as the Internet of Space Things [31,37]. This is important as it expands coverage and facilitates ubiquitous connectivity. The desirable wireless charging can be enabled by Ambient Backscatter Communication System (ABCS), explored in this section. We also compare and contrast Ambient Backscatter Communication System with the traditional Backscatter Communication System. Figure 4 gives a pictorial guide to the enabling technologies treated in this section. The challenges associated with the enabling technologies are also treated in this section. Furthermore, the challenges related to social, psychological, and commercialization issues are discussed in Section 8 of this paper.

### 5.1. Pervasive Artificial Intelligence

Over the past decade, the progress in the field of artificial intelligence (AI) has accelerated due to the introduction of deep learning (DL) and ease in its implementation [96,97]. These advancements, coupled with AI’s data processing and decision-making capability, have made it a popular tool in various fields such as wireless communication. As discussed in Section 4 of this paper, one of the key visions of 6G is to support the connectivity of billions of heterogeneous devices to the network. This requirement makes it indispensable to replace traditional mathematical models and algorithms with complex, data-driven machine learning techniques [31,47,48]. In the absence of abundant training data, the techniques in the domain of Transfer learning [98] and generative models [99] can be put to use.

Furthermore, the traditional theories cannot optimally adapt to real-time fluctuations in operating conditions [100], non-linearities, and imperfections of practical systems for which DL and Reinforcement Learning algorithms are better suited [101]. As a result of this, AI is considered one of the key enabling technologies to cater to the open problems of the 6G communication system. In this subsection, we summarize the literature pertaining to the application of AI for various open problems of 6G, indicating its pervasiveness

Machine Learning has been applied in 5G, and [102] classify some of these applications under unsupervised, supervised, and reinforcement learning. AI’s potential to improve network handover, reduce network energy consumption, predict, detect and enable self-healing network anomalies, and optimize network planning involving base-station has been discussed in [8], all of which make AI favorable for a reduction in capital and operations costs. Chen et al. [23] give an elaborate list of critical applications of AI in wireless communication and how they can be applied to other enabling technologies such as UAVs [103], VR [104], Caching and Computing [105], and IoT [106]. This presents AI as a technology that interfaces with other technologies and facilitates 6G [107]. This is important as it helps achieve user-centric wireless services presented earlier in Table 4. Yang et al. [42] presented an AI-enabled architecture for 6G networks divided into an intelligent sensing layer, data mining and analytics layer, intelligent control layer, and smart application layer. This shows AI pervasive prospect in every stage of the OSI model [24]. A holistic application of DL is proposed in end-to-end optimal design of the Physical Layer system [108,109], and optimization of energy management strategy for infrastructure and devices [42,110]. There is a plentitude of work in literature that targets narrower problem areas. We provide a bird’s eye view of these approaches in Table 8, where we have categorized them according to their relevance in the OSI model. This table reiterates the pervasiveness of AI in future 6G communication networks.

Advanced machine learning techniques can be harnessed to manage big data and algorithm-driven applications. Toward this end, Zhang et al. [97] survey how to bridge the gap between deep learning and networking. Several deep learning techniques with potential applications to networking are delineated. An exhaustive review of the current challenges and open issues in mobile and wireless networking focusing on deep learning is presented. In [111], DL is proposed as a prospective strategy to design practical mobile traffic classifiers (TC), leveraging automatically extracted features, coping with encrypted traffic, and reflecting their complex traffic trajectories. Here, different DL techniques from (standard) TC are broached. Although the mobile context declined, the latter outcome can appeal to the broader umbrella of encrypted TC tasks. Finally, the performance of these DL classifiers is critically investigated based on exhaustive experimentation.

**Table 8:** Application of AI in different layers of 6G communication networking protocol stack.

Layer	Application	Advantage of Using AI	Reference
Physical Layer	Coverage and Capacity Optimization	Dynamic sleep control of Base stations without assuming any specific model or the environment. Dynamic Fractional Frequency Reuse Strategies.	[112]
	Adaptive Coding and Modulation	Higher spectrum efficiency or better tradeoff between data rate and reliability to suit real-time channel conditions using fewer model-based approximations.	[112]
	Symbol Detection	Detect symbols in a dynamic, multiuser environment by skipping intermediate estimation tasks.	[112]
	Channel Prediction and Estimation	Improve flexibility, scalability, and generality.	[112,113,114,115]
	Channel Coding		[116,117]
	Synchronization, Localization		[118,119,120]
	Beamforming		[121,122]
Data Link Layer	Dynamic Spectrum Sharing	Make use of the dynamic environment to tune transmission parameters to improve channel throughput and user sum rate.	[112]
Network Layer	Traffic and Mobility Prediction	Improve Prediction Accuracy.	[112]
	Mobility and Handover Management	-	[123]
	Intelligent Network Management	-	[124].
Application Layer	Intelligent Caching and Content Prediction	Reduce Latency.	[125]
	Mobile Edge Computing	Slicing, Caching, Mobility, offloading.	[126,127]
Cross-Layer	Distributed Resource Allocation in Cognitive Radio Networks	-	[112]
	Interference Alignment and Caching	Improve network sum-rate and energy-efficiency.	[128]
	Mobile Social Network Optimization	Improve reliability, optimal resource sharing, reduce latency.	[128]
	End-to-end Radio Design	Optimize communication system.	[101,108,129]

#### Challenges Associated with AI

Many challenges have been identified with the application of AI in wireless networks. Getting training data is cumbersome, and there are practically no training data to work with as we have in other fields such as natural language processing and computer vision. These data require computational and processing resources, which leads to communication costs [130]. The several features of the data, with changing values, also presents another challenge as working with higher dimensionality [131] is cumbersome. The authors in [132] presented ten challenges associated with Machine Learning applications in 6G. The challenges identified are end-to-end qualified service provision, cross-layer cooperation, dynamic online learning with proactive exploration, scalability, efficient dataset generation, computation overhead deployment, feasibility verification, standardization, learning efficiency, and deployment in distributed and centralized systems. The authors in [44] also identified challenges: Training issues, lack of explainability, interoperability and bounding performance, and uncertainty in generability.

Deep-learning-based solutions require high computational complexity, which might not fit in current mobile phones [109]. Barring the complexity, Artificial Neural Network (ANN) based RL algorithm must be meticulously designed to reduce computational resources required on these devices [23]. Quantum communication [26] offers a promising approach to circumventing the challenge of limited computational resources and energy efficiency [26]. Applying Artificial Neural Networks in IoT also comes with the trade-off challenge between accuracy and computational/energy requirements [23]. Thus, identifying the right use cases for ML/DL applications is more of a priority than tweaking neural networks and network procedures [133].

### 5.2. Reconfigurable Intelligent Surfaces

**Figure 5:**
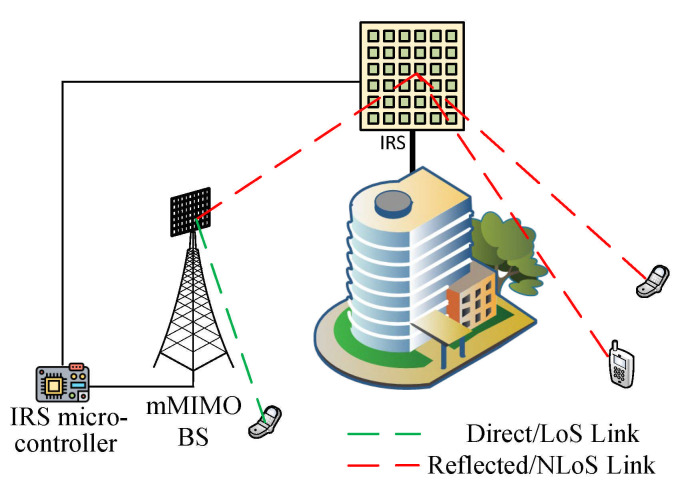
This diagram shows a Reconfigurable Intelligent Surface (RIS)-assisted communication system.

Reconfigurable Intelligent Surfaces (RIS) [107] are envisaged as a key enabling technology in 6G, just like massive MIMO in 5G [17]. They have been proved to improve the performance of networks [134] and with virtually no interference [135]. They are envisioned to facilitate energy efficiency because they are relatively passive and only require a limited number of active antennas at the BS to achieve massive MIMO gains [95]. The advantage of RIS is not just them having passive elements that improve energy efficiency. They also have a low-cost compared to other alternatives. They have name variants such as large, intelligent surfaces [136,137], reconfigurable intelligent surfaces [18,138], and software-controlled metasurfaces [139]. Figure 5 shows an RIS-assisted communication system with the RIS reflecting signal, thus enabling communication from the BS to the mobile unit.

The metasurfaces are implemented with conventional reflect arrays [140,141], liquid crystal arrays [142], or software-defined metamaterials [139,143]. Table 9 juxtaposes the types of metasurfaces, classifying them based on structure, energy consumption, and more. The authors in [138] posited that RIS improves energy efficiency as they do not require power amplifiers, which contrasts with other relay systems. Consequently, the RIS ameliorates the problem of interference in an ultra-dense network since the RIS is passive [67]. Being passive also makes it easy to be deployed, and they can be deployed on buildings and structures. It also can be configured to provide security at the physical layer [67,144,145]. The RIS also supports low-power device-to-device (D2D) communication, which helps to actualize Simultaneous Wireless Information and Power Transfer (SWIPT) [67,146]. We compare the features of RIS with existing relay systems in Table 10.

The spectral and energy efficiency can also be enhanced by jointly optimizing the active transmit beamforming at the AP and the passive reflects beamforming at the RIS. Therefore, RIS improves spectral efficiency [138]. Operating passively, RIS help reduce the implementation cost of future wireless communication [87,135]. In addition to passive beamforming, which provides communication between the user and BS, RIS use in information transfer through sensors has also been proposed [137,147]. According to the authors in [138], other advantages of RIS are: they are more energy-efficient and environmental friendly compared to conventional relay systems and compatible with standards and hardware of earlier wireless generations. RIS is also being considered to be deployed to secure wireless networks [148,149,150].

**Table 10:** Contrasting RIS with similar existing technologies for 6G.

Features	RIS	Active, Intelligent Surface-Based Massive MIMO	Backscatter Communication	Amplify and Forward Relay
Hardware architecture	Passive elements	Active elements	Active elements	Active elements
Power consumption	Low	High	High	High
Environment propagation control	Adjusts the phase shift through smart controllers.		Reflects received signal from external sources such as TV.	
Noise effect	Only reflects the received signal.	The use of artificial noise is helpful to the system [151].	The backscatter signals are weak and with a low signal-to-noise ratio.	Amplifies received signal and receiver noise.
The unit cost of deployment	Low	High	Low	Low

#### Challenges with Reconfigurable Intelligent Surfaces

Although the RIS looks promising to facilitate communication beyond 5G, some challenges have been posed. In [152], the limitation in the phase range of unit cells making up the aperture was identified. Achieving a full-phase range may require complex cell topology, which would increase hardware complexity. Increasing hardware complexity defeats the aim of energy efficiency proposed in 6G networks. Another challenge identified is the quantization of the phase range of the unit cells. The flexibility of RIS is also desired so it can adjust to the dynamic characteristic of the reflected wavefront.

Dynamic modulation of RIS using low-power semiconductor elements such as PIN diode was proposed to address dynamic reconfigurability problems [152,153]. This was implemented in [67], and a variable resistor load was also proposed to control the reflection amplitude. However, controlling the reflection amplitude and phase shift is costly in practice because it is better to implement discrete amplitude/phase-shift levels that require a small amount of electromechanical systems, i.e., microelectromechanical systems (MEMS) [135] have made reconfiguring easy, through controlling phase shifters in real-time.

Another challenge associated with the RIS is estimating the channel information [135,154], correctly. Most of the existing works have been based on the conjecture that RIS has perfect channel state information. This has not been proved to be true and highly unlikely in practice because the RIS does not have radio resources for channel estimation [155], although equipping the RIS with a low-power RF chain for channel estimation capability has been proposed [67]. However, this compromises the reduced complexity and energy-saving features desired in 6G networks. Ironically, RIS can be deployed to allow energy transfer [156]. The high-dimensional channels are estimated in severe under-sampling constraints [152], making accurate estimation of Channel Information challenging. Consequently, it is difficult to actualize the RIS phase control due to imperfect channel estimation and hardware complexity [87].

Models have been proposed to resolve the challenge of channel estimation, one of which is the Ray-tracing-based methods [157], which were proposed to estimate the channel information by the authors in [18]. However, they are costly and require stringent site information [67]. In [152], the knowledge of signal covariance matrix through extensive research in random matrix theory and high-dimensional statistics was proposed. This corroborates the dependence on basic sciences as suggested in [40]. The authors in [158] proposed a three-stage mechanism for channel estimation: sparse matrix factorization, ambiguity elimination, and matrix completion, respectively. The methods used were based on mathematical tools of random spatial processes and stochastic geometry. The theoretical framework used was validated with Monte Carlo Simulation. A minimum mean squared error (MMSE) based channel estimation protocol between the BS and RIS to serially estimate each RIS link was proposed in [155]. Machine learning, Deep learning, and Federated learning models have also been proposed to help resolve this challenge at a lower computational cost [159,160,161].

A deep reinforcement learning model was proposed in [162] to predict the RIS reflection matrices with minimal training overhead. A joint compressive sensing and deep learning solution were also proposed in [159]. This designs the LIS reflection matrix with negligible training overhead. The idea presented in the paper, however auspicious, was proved in theory but not assessed with practical data. Further research is needed in researching this with practical implementation. Although this is a daunting task as Machine learning methods are not error-free, getting training data sufficient for deep learning models is quite cumbersome [163]. Nevertheless, this is a viable research area to explore. It is imperative to model the channel with real practical data to assess its accuracy and compare it with existing channel estimation models that have been deployed.

### 5.3. UAVs/ Satellite Communication

Unmanned Aerial Vehicles (UAVs), also known as a swarm of self-governing drones [164], are aircrafts without a human pilot onboard the flying network (thus the name “unmanned”) [165]. It is supported by a control system and a human who controls it remotely [166]. They are usually deployed in military applications [167]. Other applications in traffic monitoring [168,169], fire detection [167,170], maritime services, filmmaking, journalism [171], smart farming, remote surveillance, business and industry, and more have been seen over the years. We believe UAVs are key in actualizing future wireless networks, and this has been corroborated by various researchers [172,173].

**Figure 6:**
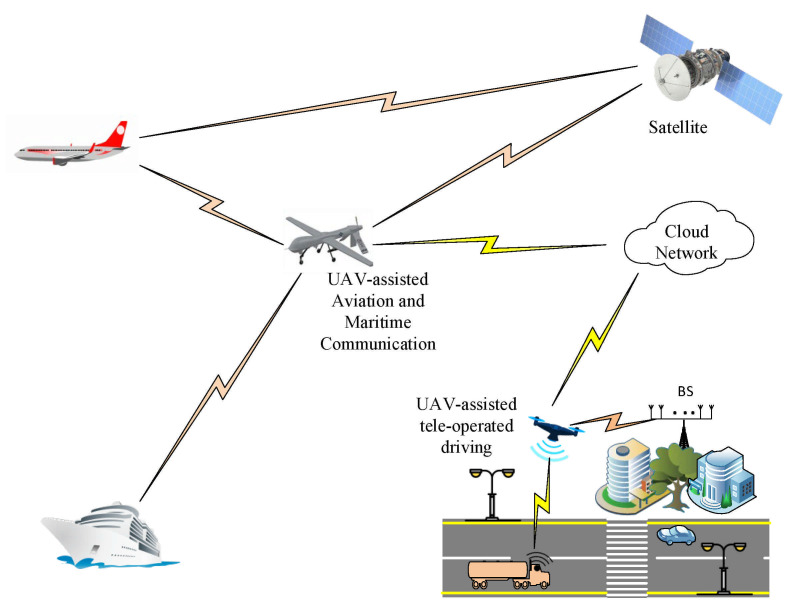
UAV/CubeSat facilitated ubiquitous connectivity.

The Internet of Space Things, comprising of CubeSats and UAVs, is also proposed in [31] to enable key technology for 6G and beyond. CubeSats, which are also known as U-class spacecraft, are miniaturized several spacecraft with sizes which are multiples of U, up to 6U, and U being 10×10×10 cm cubic units. They have a lower unit cost [31] as they weigh less [38] and, in most cases, share a rocket with a larger satellite [38]. CubeSats are currently deployed in earth sensing [174,175], positioning [176], IoT [177], machine-to-machine communication [37]. Other applications to science and commerce are treated in [178]. Figure 6 shows how UAV and CubeSats can enable ubiquitous connectivity.

#### 5.3.1. Applications of Unmanned Aerial Vehicles

Several application scenarios for Millimeterwave-empowered Unmanned aerial vehicle (mmWave-UAV) such as access point, communication terminal, and backbone link are investigated in [179]. The work demonstrates the coupling relationship between mmWave beamforming and UAV positioning for optimal mmWave-UAV communication. Furthermore, the key enabling techniques for UAV communications, joint Tx/Rx beam alignment, beam tracking, multi-beam forming, full-duplex relaying, and the potential challenges are discussed extensively. Remote Controlled Unmanned Aerial Vehicles (RC-UAVs) are envisioned to aid heterogeneous wireless communication [180]. Cooperative game theory was deployed to select the best UAV during the handover process and optimize handover among UAVs by reducing; handover latency, end-to-end delay, and signalling overheads. Further, Software Defined Network with Media Independent Handover UAV (SDN-MIH-UAV) architecture was employed as forwarding switches to achieve seamless mobility.

The flying networks can be deployed to provide internet services to disaster-struck areas. It will facilitate the ubiquitous connectivity being desired in 6G networks. It is one of the major enabling technologies for achieving ultra-low latency communication [24]. UAVs also have a more robust line of sight than fixed BS and degree of freedom controlled by mobility. This is an ongoing development in harnessing the enormous potentials of UAVs for wireless communication. UAVs can withstand geographical and environmental limitations on wireless communications such as ships on the ocean. Facebook is partnering with Airbus to make drones that beam the internet from high altitudes. This helps to provide wireless connectivity in rural areas. A similar trend is Google deploying Loon in some rural communities. However, this uses balloons and not UAVs. UAVs are different from Satellite communication as they operate at a much lower altitude. The swarm of self-governing drones also possesses flexibility and can be used to collect, deliver, and transmit telematics. They also help with mobility and handover management in wireless networks [42,165].

#### 5.3.2. CubeSat Communication

Satellite communication will be key in actualizing 6G networks, and miniaturized or small satellites are being developed. Small satellites have a relatively low design and deployment cost. They also have lower implementation complexity. These satellites are deployed in Low Earth Orbit (LEOs), which allows them to have a low latency communication. LEO satellites are proposed in [82] as the most prospective satellite communication. For example, SpaceX, Facebook, OneWeb, and recently, Amazon are the key players who have existing LEO satellite projects for internet-beaming [181].

There are different satellites based on their weights; pico-satellites, also known as CubeSats, have emerged as the most popular [182]. However, most of the CubeSats research focused on remote-sensing applications and not much consideration on communication applications. The authors proposed the Internet of Space things in [37], and CubeSats will be a powerful technology to facilitate this. Additionally, it is worthy to note that some literature has expanded space communication to include high altitude and Geostationary Orbit Satellite (GEO) [183,184]. Table 11 compares UAV and CubeSat communication, comparing the altitude, control, dimensionality, and power control.

#### 5.3.3. Challenges with UAV/Satellite Communication

The authors in [185] identified some challenges with UAV-enabled Wireless systems, such as the topology of the network changing with the nodes and the links altering. The routing protocol requires a complex implementation; maintaining user sessions with the intermittent transfer from an out-of-service UAV to an active UAV and energy conservation in UAVs also pose challenges. UAVs have limited communication resources, nodes are not fixed, and the channel can be impaired. Therefore, the challenges with UAVs can be categorized as challenges with resource management [186], resource allocation, routing, and energy constraints. Applications of AI-controlled UAVs have been suggested to solve these issues [24], and deep reinforcement learning is proposed to enhance handover amongst several UAVs [42].

Building the UAVs to work in different environments by enhancing the capability of the networking protocol helps to address the issue of intermittent network changes and channel impairment [185]. Optimizing interactions between nodes, such that a node can go to sleep when they are redundant, reduces power consumption and, subsequently, the energy requirement of the UAVs. Overlaying UAV-to-UAV (U2U) connection and cellular ground user uplink (GUE-UL) communication was suggested by [29] as a better alternative in urban areas for simultaneously maximizing GUE-UL performance and guaranteeing a minimum U2U coverage rate of 100 kps to the majority of UAV pairs.

One of the significant challenges with CubeSats and other miniature satellites is the lack of a standardized channel model [187]. Some models have been presented, such as RF and optical links, but the channel presents signal attenuation because of molecular absorption and scattering losses by gas molecules and aerosols in the atmosphere. Additionally, background noise and the stars, beam divergence loss due to beam diffraction close to the receiver, atmospheric turbulence, and pointing loss attributable to satellite vibration [182]. As noted by the authors in [37], other challenges with CubeSats are long delays, signaling issues, and topological variations. There are also challenges with integration with next-generation networks, data scheduling on account of the limited transceiver. A deep learning approach was proposed in [96] to resolve resource allocation challenges in CubeSats.

### 5.4. Terahertz Communication and Optical Wireless Technology

RF frequency band below 6GHz has been exhausted, and there are research interests in higher spectrum. Spectral efficiency can be increased by increasing bandwidth while applying massive MIMO. This is achievable at higher frequency bands (millimeter-wave, Terahertz, and Free Space Optics). The authors in [188] proposed the opportunity for terabit-per-second data rates, high energy efficiency, and miniaturized transceiver size. Transmitting at higher bandwidth also makes an application such as Holographic communication, which requires very high data rate, permissible. Akyildiz et al. [69] identified some THz communication applications obtainable at the macroscale, microscale, and nanoscale. There has been development in a chip-wireless network on chip (WNoC)-for THz band transmission [189]. However, characterization of the channel has been a challenge that casts doubt on the practicability.

Rappaport et al. [12] also identified wireless cognition, hyper-active position location, sensing, and imaging as some of the applications obtainable when transmitting above the 100 GHz range. Optical Wireless Technologies such as light fidelity, visible light communication, optical camera communication, and Free-Space Optical (FSO) communication have been used since the 4G. They provide a high data rate, low latency, and security [48]. VLC is a good cost-effective means of alleviating the challenge of spectrum shortage in the sub 6 GHz band. It provides a higher frequency spectrum and dual utility for light bulbs: illumination and communication [190]. It makes communication achievable wherever light bulbs are being used, thereby facilitating the ubiquitous connectivity expected in 6G networks [191].

Furthermore, they also facilitate underwater communication [192]. VLC possesses high bandwidth, and it is immune to interference from other electromagnetic sources [15]. VLC also provides system security [193], and it is expected to break the terabit-per second barrier [6]. FSO allows broadband communication through the transmission of modulated light signals through free space [194]. This provides a cost-effective alternative communication for a low-income population [195]. It can be deployed in terrestrial and inter-satellite communication [16]. Table 12 compares THz and other spectrum bands such as mmWave, VLC, and FSO.

#### Challenges with Terahertz Communication and OWT

THz communication has inherent short propagation capability. It becomes difficult to detect weak signals as sensitivity decreases at the THz range [198]. At higher frequencies, bond cables suffer signal degradation. As presented by the authors in [8], communication in the THz range is susceptible to severe path-loss and atmospheric absorption, hardware compatibility, waveform, channels, and protocols. The authors in [152] also presented three challenges with transmitting at a higher frequency band: packaging and interconnect techniques that provide reliable interconnection between interior and exterior environment, transceiver design that meets the compact physical size power efficiency, and issue with standardization and measurements [199]. They suggested cutting-edge technology in micro-machining, low-temperature co-fired ceramic (LTCC) [200], and additive manufacturing techniques in improving packaging challenges. Spatially over-sampled antennas and phased array architectures can be leveraged to improve the transceiver design, and electro-optic sampling can solve the issue of measurements. Spatially over-sampled antenna, new phased-array antenna, and compact computational approaches also are promising in actualizing accurate beam steering with less exponential power [12].

Yifei et al. [49] also identified the following challenges with THz communication: severe path-loss, weak diffraction effect at short wavelength, excessive attenuation from the resonance of molecules in the air, and weak diffraction that result in sensitivity to blocking and shadows, superfast channel fluctuation. Zhai et al. [201] proposed *THzphism,* a frequency-based beam spreading technique that utilizes several true-time delay (TTD) devices in a phased array antenna. This helps to improve angular coverage while maintaining the distance covered by the THz system. This encourages the adoption of phased-array-based beamforming in reducing severe pathloss. Precoding techniques such as analog beamforming, hybrid precoding, and delay-phase precoding were also proposed by the authors in [202] as providential in reducing the severe-path loss challenge experienced in THz transmission.

Akyildiz et al. [69] considered the challenges associated with THz band device technologies and THz band communication networks. They also identified security as another challenge of THz band systems. Petrov et al. [188] identified the following challenges associated with THz transmission: design of supporting THz electronics, THz channel modeling [187], coverage planning, effective medium access control, and support for nodes mobility. Ideally, a photonic solution would be a viable option for transmission in THz [49,203], but it has a larger component size [45]. Plasmon-based THz link components have also been promising in facilitating THz communication as they have miniaturized sizes and operate at ultra-high data rates [45].

Hybridization of micro-LED matrices and CMOS driver arrays on a single chip will also be required to solve the challenges of huge matrices [6]. As the Doppler effect is more severe in the THz range and channel status is always changing, spatial consistency is proposed to give realistic and updated CIRs [12]. The authors in [6] also presented the need for the complex device by parallelizing several chips and introducing a dedicated optical imaging system leading to optical beamforming, which will significantly increase cellular throughput. A CMOS-based modulation circuit was proposed in [41] to get an improved performance gain.

VLC is posed with the challenge of interference with ambient light sources, and it is proposed that increase in VLC devices will result in interference between VLC devices [15]. Signal jamming [204] and data snooping are also some of the security challenges with VLC that need to be resolved [193]. Handover management, configuration algorithm for both illumination and configuration, and compatibility with existing spectrums are challenges identified with VLC [190]. LED transmissions provide a short-range susceptible to absorption, shadowing, and beam dispersion [191]. VLC and FSO require line of sight for communication. Non-light sight communication attributable to obstruction on the beam path makes communication difficult. There is also doubt on the safety of lasers [194]. Just as VLC, FSO is susceptible to absorption, scattering, scintillation, atmospheric turbulence, and other hostile environmental conditions [195,205]. Geometric loss, background noise, and misalignment error are other challenges of FSO identified in [86].

### 5.5. Cell-Free Massive MIMO

**Figure 7:**
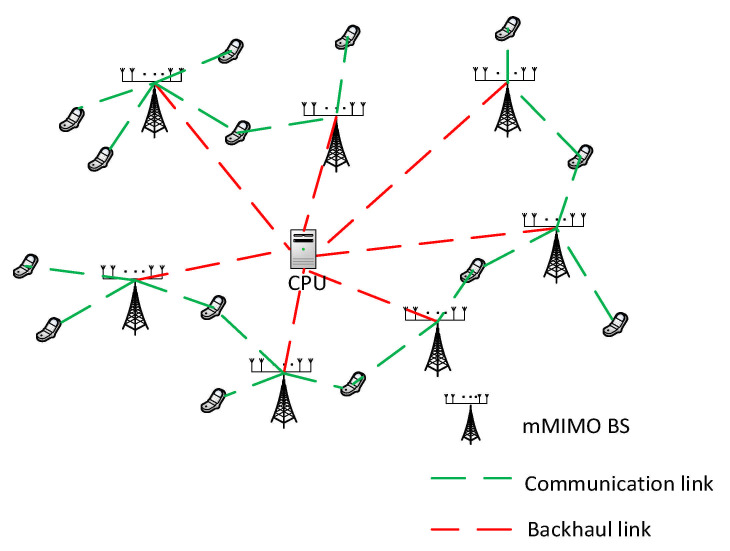
An illustration of Cell-free Massive MIMO.

The combination of Time Division Duplex (TDD), massive MIMO operation [206], densely distributed network [207], and user-centric network [208] refers to the ubiquitous cell-free massive MIMO [22]. Cell-free Massive MIMO, which is the generally adopted terminology, combines the distributed MIMO and Massive MIMO [68] concepts such that there are no boundaries and cells in the network. This allows users to move seamlessly from one network to another. It cancels out interference problems and reduces the frequent handover experienced in current networks, thereby improving user experience. Figure 7 shows a pictorial layout of the Cell-free Massive MIMO communication system.

Cell-free Massive MIMO has significantly outperformed existing small-cell systems throughput and more resistant to shadow fading correlation [68]. Massive MIMO was proposed by Alamu et al. [209] to improve Energy Efficiency and Spectral Efficiency. Utilizing spatial multiplexing in Massive MIMO allows multiple users to share spectrum resources efficiently. Cell-free Massive MIMO presents high spectral efficiency, system scalability, and almost-optimal linear processing [22]. It can also meet the requirements of ubiquitous, ultra-reliable low latency communication desired in 6G networks. The potentials of larger intelligent surfaces (LIS) in Massive MIMO operations have been identified in [20] and also in LEO satellite communications using full frequency reuse (FFR) [210].

#### Challenges with Cell-Free Massive MIMO

One major challenge with cell-free massive MIMO is obtaining accurate Channel State Information (CSI) [22,133]. There are also challenges with practical deployment. There is cost/complexity of the deployment, limited backhaul capacity [211], network synchronization, and a cost-effective radio stripe system presented in [22]. Issues such as practical control, distributed signal processing, resource allocation, channel modeling [187], and estimation were raised [22]. There have been efforts to circumvent this challenge of channel estimation. The authors in [133] proposed Machine Learning (ML) as a primary tool for channel estimation and channel charting through unsupervised learning as a promising future direction. However, concerns about active eavesdropping have been raised [212], which casts doubt on the security of these systems.

To improve security, Hoang et al. [212] suggested maximization problems to maximize the achievable secrecy rate subject to the quality of service constraints. There may be a trade-off problem between secrecy rate and energy consumption in future cell-free massive MIMO networks. Matthaiou et al. [152] identified the following challenges with the cell-free Massive MIMO: practical user-centric approaches, scalable power control, and the need for advanced distributed signal processing (SP). These are areas that need research focus.

### 5.6. Blockchain and Distributed Ledger Systems

Many papers addressing the vision and requirements have proposed blockchain adoption in 6G wireless networks [47,81]. Blockchain is proposed to provide network decentralization [6,40], spectrum sharing [9,81,95], and security [41,45,48]. The authors in [47] presented blockchain as one of the applications of 6G. Blockchain is a form of distributed ledger [48] that serves as a database with several nodes replicating and saving an identical copy of the ledger. It is a perfect complement to the massive IoT with improved interoperability, security, privacy, reliability, and scalability [32,213]. This makes it a good enabling technology to improve the integrity and security of future wireless networks.

Blockchain is also being considered for the fledgling 5G communication network. Blockchain was proposed to give 5G IoT integrity, accelerated data change, lower cost, security, immutability [32]. Integration of machine learning with blockchain in 5G and beyond has also been proposed [28]. Blockchain provides real-time data delivery, resilience, and low latency to the network [214]. The integration of edge computing with blockchain is also a desirable research field. This provides reliable access and control of the network, storage computation over a large number of distributed edge nodes, and improved network security, data integrity, and computation validity of the system [215]. Jocovic et al. [216] proposed integrating Interplanetary File System (IPFS) and blockchain to provide enormously high security, transparency, credibility, and immutability of stored data. The application of Blockchain in UAVs is also being considered [28].

#### Challenges with Blockchain Technology

Although blockchain technology is proposed to give security and privacy to future wireless networks, the system on its own is not resistant to cyber-attacks. Security and privacy have been identified as concerns in blockchain technology [217]. Xie et al. [217] also identified a limited throughput, energy efficiency, lack of incentive and punishment mechanism and regulation as some of the challenges associated with blockchain application in smart cities, enabled by wireless communication. Scalability has also been identified as a challenge in blockchain deployment [28,215].

Yang et al. [215] also identified research challenges with integrated blockchain and edge computing systems such as scalability enhancement, security and privacy, self-organization, function integration, and resource management. The integrity provided by the immutability of the blockchain comes at the expense of higher computational capability and delay performance. This can be resolved by applying the edge cloud [79]. Big Data can also be adapted to perform pattern recognition on ledger transactions to give more security. AI can also be leveraged to design a new generation blockchain that supports automatic generation of smart contacts, enhanced security under malicious attacks, and highly flexible operations [215].

### 5.7. Ambient Backscatter Communication

Ambient backscatter (AB) was proposed by [6] to enable tiny devices to operate without a battery, such that they are powered by redirecting ambient RF signals without requiring active RF transmission. Ambient backscatter communication is a form of backscatter communication that can harvest energy from devices in its “ambient” environment such as Wi-Fi, TV, and RF signal to modulate information [218]. When this passive backscatter system is integrated with an active transmission system, it forms the symbiotic radio (SR) [219]. This addresses the energy efficiency problems in low-power consumption devices such as sensor networks. This helps to reduce the cost of devices with low-power components and reduced infrastructure [31]. There is also no need to allocate a different frequency spectrum for ambient backscattering communication systems (ABCS), thereby improving spectrum resource utilization [27]. When perfected, ambient backscatter communication will enhance the battery life of mobile devices [218].

Ambient Backscatter Communication (ABC) sensors were deployed to solve the problem posed by the fusion of local decisions over multiple-access fading channels in WSN, considering the IoT vertical [220]. ABC sensors are deployed to detect the presence or absence of a given phenomenon and transmit their decisions to a multiple-antenna fusion center reader (FCR). In this case, a part of an incident RF ambient signal is reflected. This facilitates the development of decision fusion rules in the full and statistical channel state information (CSI) scenarios to enhance their implementation and lower computational complexity. To increase the data rate of passive transceiver-to-transceiver communications for ambient backscatter (AB), Qian et al. [221] employed *M*-PSK for backscattering and derived the optimal multi-level energy detector and the closed-form symbol error rate (SER). A 4PSK-AB hardware prototype that gives a data rate of 20 kb/s was developed to integrate the 4PSK-modulated transmitter, multilevel detector, and energy harvester. Recently, the issue of symbol detection in ABCS with the multi-antenna reader was addressed [222]. Leveraging the ON-OFF keying modulation, the optimal detector, which can avoid the error floor phenomenon, was devised. Additionally, multiple antennas are employed at the reader to improve the bit error rate (BER) [223,224] and extend the tag–reader communication range. Table 13 compares ambient backscatter communication with traditional backscatter communication.

#### Challenges with Ambient Backscatter Communication

There is a limited coverage area for operation as backscatter transmitters need to be placed near their RF source [27]. This means that one has to be close to the backscatter transmitter to utilize the system. This limits mobility while retaining communication. In [27], the authors also identified reduced transmission performance on account of interference between transmit and receive antenna thanks to proximity and the dynamics of ambient signals, data transmission scheduling for backscatter devices as challenges. ABCS are also susceptible to security threats [27].

Liu et al. [225] identified at least three challenges associated with ambient backscatter communication, namely: the challenge of extracting backscatter information owing to weak signals, the need for less-complex digital signal processing techniques, and hardware architecture need for a centralized controller to control communication. Challenges associated with EE and SE and compatibility with other wireless communication systems have also been identified [31].

### 5.8. Quantum Communication

One question posed by the authors in [133] is what happens when massive MIMO is combined with quantum communication or molecular communication? There have been growing research interests in quantum communications and how they can be adopted to B5G networks. Quantum computing gives high data rates and security [41]. Quantum communication [226] was proposed as enabling architecture in 6G as it has the potential of accelerating the speed of information processing leading to a more optimal solution in 6G communication [95]. Quantum communication may also provide a shield from cyber-attacks through sophisticated quantum computing and quantum communication [30].

Future quantum internet is proposed to be likely based on long-distance quantum optical communication suitably interfaced with solid-state nodes for quantum information processing [227]. There are also interests in quantum-based satellite communication [228] and quantum machine learning and deep learning for 6G [229]. Quantum computing can significantly accelerate and enhance AI algorithms that require big data and massive training [81]. The concept of quantum optical communication opens up innovation in telecommunication and ICT, and quantum optics is a providential paradigm for the transmission and control of optical signals to have a better degree of freedom [26]. The authors in [26] delineated the infusion of quantum and THz communication in the actualization of 6G.

#### Challenges with Quantum Communication

While channels of classical computing are well treated within the framework of classical information theory, the quantum channel is not entirely understood [229]. Constraints identified in quantum internet by authors in [230] are quantum measurement, entanglement, non-cloning, and teleporting. Getting supporting infrastructure for quantum communication with quantum switches and routers becomes difficult as a result of the non-cloning theorem [229]. Cacciapuoti et al. [230] also identified decoherence and fidelity, entanglement distribution, and deployment challenges as some of the challenges associated with quantum communication. Blockchain has been suggested to enable security and privacy in quantum communication [28,217].

Table 14 compares these enabling technologies and how they facilitate the requirements desirable in 6G.

## 6. 6G Applications

Every generation of wireless networks has facilitated new applications, and 6G is not an exception. The need for higher data rates, lower latency, high reliability, and more have given rise to the development and deployment of new wireless generation networks. In this section, we examine the applications to be facilitated by 6G. Although these or similar applications have been proposed to be enabled by 5G, 5G cannot meet the requirements to facilitate these applications seamlessly at the current development stage. Some limitations in 5G, such as scarce bandwidth and high-energy consumption, have been identified [231]. Although 5G tests have shown prospects with the actualization of a data rate of 8 Gbps [232] and 1 Gbps at 6.5 km distance [233], these are still below the peak data of 20 Gbps proposed in the literature. Therefore, existing 5G networks cannot meet high-speed intensive applications such as holographic communication, which require 1 Tbps for seamless communication [39]. Other limitations identified with 5G wireless networks are high interference due to massive interconnection, insufficient computing capacity, and lack of ubiquitous connectivity. Hence there is a need for 6G communication, which promises better features than 5G.

Some of the applications to be facilitated and fully enabled by 6G are holographic communication, teleoperated driving, tactile internet, Industry 4.0, and more. New applications that were not considered in the 5G context, such as IoBNT and Digital replica, are also introduced in this paper. Table 15 gives a summarized description of these applications. Furthermore, we outline the challenges in achieving some of these applications. Additionally, desirable features of these applications are considered. These applications, the 5G limitations, and how 6G wireless networks will enable them are highlighted in this section. In this literature, it has been established that the existing features of 5G are not efficient enough to enable the requirements of these technologies. We consider each application in this section, pointing out some of the requirements that make 6G an ideal candidate for these applications.

### 6.1. Holographic Communication

Holography utilizes a laser beam to produce images. This concept has been used in color motion holography [234], metasurface holography [235], 3D holographic display [236], and holographic type communication (HTC) [237,238]. New 5G enabled disruptive technology is used in persuasion for real-time holograms [239]. An exciting feature of 5G technology is hologram generation and delivery [240]. Timing synchronization setting for transmission jitter of less than a microsecond is required in multi-stream holographic applications. More reliable remote surgery needs latency of less than 1ms, which is extremely difficult to achieve in the current 5G systems [6,241]. Although some promising results have been discovered in 5G research, 1Gbps was achieved over a 6.5 km distance, giving a world record [233]. This has been experimented with in the 26 GHz range. However, this is still way below the requirements needed to enable full holographic support in the order of 1Tbps [39]. Therefore, the major challenge with holographic communication is high data-rate and low latency [10]. To achieve seamless holographic communication, extremely high data transmission, hundreds of times more than what is obtainable in 5G systems, is required [8].

With 6G wireless networks proposed to have a data rate in the order of 1Tbps and ultra-low or non-existing latency, holographic communication will cease to be just a utopian technology limited to movies. As we go higher in the frequency spectrum, and with technologies such as VLC and more, higher data rates would be achieved. A truly ubiquitous environment empowered by real-time holograms could make smart devices appear like horse carriages. Peak data rates would reach Tbit/s in the 2030s, which will require massive bandwidths availability. The huge data rate would enable 16K video resolution in 360 degrees with a refresh rate of 240 Hz required for holographic displays [242]. 6G is expected to meet these requirements to support the massive transmission of real-time data over the air. Furthermore, 6G-empowered XR technologies and holograms supported by advanced AI techniques would help clear the time and space barriers to advance remote surgery [240].

Microsoft developed Holo Lens, and this has stirred more research interests in this field. Projects such as Holoportation, Ada, and many more, cutting across engineering, commerce, games, etc. Spatial, a virtual communication application that uses Microsoft Holo Lens 2. This allows workers in different locations to communicate using avatars or holographic shapes of themselves. An effective holographic video conferencing enhances the borderless workplace experience where people can work from home. The holographic display has also been primarily embraced in recent times. The holographic display market is projected to reach USD 3576 million by 2020 from USD 567 million in 2013 [243], USD 7.6 billion in 2023, and projected to nearly USD 1 trillion in 2030.

### 6.2. Teleoperated Driving

6G is proposed to increase the functionality of Autonomous Driving. There is interest in teleoperated driving (or semi-autonomous vehicles) where a human controls the vehicle remotely. Teleoperated driving has been used in deep-sea and space exploration. Companies such as Ericsson and Huawei have tested teleoperation on 5G networks [244]. Teleoperated driving will require ultra-low latency that communicates signals and instructions between the driver and the vehicle, especially in the face of danger where an immediate response is needed. This, if achieved optimally, will enhance car-rental services of the future. A high level of security, privacy, and network integrity is also desired. Although research is towards fully autonomous vehicles, teleoperation is desired when autonomous mode fails or a complicated scenario requires human intervention. Autonomous vehicles are being considered under the scope of the emerging 5G [245]. However, the 5G at its current development state cannot meet the actual requirements of a fully developed 5G wireless network. Thus, the need for 6G wireless network development.

### 6.3. Tactile Internet

Tactile internet enables haptic interaction with machines, giving rise to visual feedback and robotic control. This facilitates efficient human-to-machine interaction, machine-to-machine interaction, with key examples found in industry, robotics and telepresence, virtual reality, augmented reality, healthcare, road traffic, education and culture, and smart grid [246]. This technology is useful where human presence is needed to carry out a task [39]. A valuable application of tactile internet is in telesurgery, allowing surgeons to perform surgery remotely [247]. The major challenge with tactile internet is having a seamless network and a delay that humans do not perceive. To achieve this, low latency of 1 ms is required- this is the primary requirement for tactile internet. This was projected to be achievable in 5G. However, tests have shown existing 5G networks to have a latency of 10–16 ms. Therefore, it is expected that 6G wireless networks, which are proposed to have a latency of less than 1ms, will enable tactile internet and haptic communication. Additionally, precision with very high accuracy is desirable [164] and will be facilitated by 6G. Another desirable feature of tactile internet is security; blockchain, a proposed enabling technology for 6G, has been proposed to allow for a trusted, secured, and reliable tactile internet architecture [217].

### 6.4. Internet of Bio-Nano Things (IoBNT)

Research advances in nanotechnology and communication engineering have engendered embedded computing devices based on graphene and metamaterials. Although artificial materials (i.e., synthesized materials, electronic circuits, etc.) have been developed, a more biological application is preferred using biological cells and interfacing the bio-chemical interaction with the electrical domain. This is to lower the risk of exposure to toxicity associated with artificial devices. These devices can perform intra-body sensing, actuation, processing, and networking with a small size and implanted. They have a low data-storage capacity and low transmission range [39]. The Internet of Bio-Nano Things is proposed to be applied in health care, military, and security fields [248]. The authors in [31] identified IoBNT to aid early diagnosis of diseases, especially in the face of the COVID-19 pandemic. However, doubts that this technology might not be ripe enough for 6G have been identified [31].

Nevertheless, it will play a crucial role in future wireless networks. To enable this, challenges associated with IoBNT need to be resolved. Embedding with molecular communication (MC) is proposed to improve the range of transmission [248]. However, challenges exist in the design and development of nanodevices and coordination of molecular communication in nanodevices [249]. Additionally, there are challenges with the security and bioethics of this technology [248]. Furthermore, challenges with modeling the peculiar non-linear biological environment exists.

### 6.5. Industry 4.0 and Beyond

The fourth industrial revolution, also tagged Industry 4.0, is expected to enhance the computerization obtained in the third industrial revolution. Although 5G initiated Industry 4.0, 6G is expected to facilitate its full actualization. A combination of cyber-physical activities, IoT and IIoT, cloud computing, and AI will make Industry 4.0 achievable [250]. Some technologies that will transform Industry 4.0 are Big Data and Analytics, Autonomous Robots, IIoT, Cybersecurity, AR, Cloud, and more. High-precision manufacturing is vital to the actualization of Industry 4.0 [6], as it minimizes human intervention with no trade-off to accuracy. This requires high reliability and extremely low latency; mMTTC and URLLC, which will be improved in 6G, makes the actualization of this possible. Industry X.0 was introduced in [31] as a succession to Industry 4.0, and 6G sets a foundation upon which this would be built. The role of big data [251] and biology [252] in industry 5.0 has been investigated. Industry 5.0 is proposed to be user-centric and personalized service-oriented [39].

### 6.6. Multisensory XR Applications

Extended reality (XR) is a term that combines VR, AR, and MR (mixed reality) [253]. It takes application in medicine, education, entertainment, and other fields of life. Research is progressing towards the actualization of full Immersive XR. A new concept of Quality of Physical Experience (QoPE) that merges human factors from the user with classical QoS and QoE was presented by the authors [47]. This facilitates the multisensory feature desired for a more user-centric experience. The main challenge, which was also pointed out in [8], is inadequate hardware capability and insufficient wireless capacity. Table 16 compares the AR, VR, and MR, which make up XR.

### 6.7. Blockchain/Distributed Ledger Services (DLS)

Blockchain has been identified earlier as one of the enabling technologies for the actualization of 6G. 6G also provides an enhanced blend of massive machine type communication (mMTC) and URLLC [255]. The reliable connectivity and ultra-low latency ensure the efficient running of DLS, thereby making the relationship symbiotic [47]. Blockchain in 5G and beyond has been explored in [256] to enable emerging mobile services.

### 6.8. Connected Robotics and Autonomous Systems (CRAS)

The CRAS concept was proposed as an application of 6G in [47,48]. CRAS is being developed, and systems such as autonomous cars, drone-delivery systems are gaining traction. This is applied in manufacturing and industrialization processes. CRAS requires high reliability, ultra-low latency, and high data rate that existing 5G networks cannot provide at this stage of development. 6G networks are expected to meet these needs.

### 6.9. Wireless Brain–Computer Interaction (BCI)

Wireless Brain–Computer Interaction (BCI) devices help support, enhance and generally improve human functions [257]. This is mostly used in health care to aid people with severe motor disabilities [258]. This requires ultra-low latency and perceptual services that 6G is expected to provide. This is especially very important in non-visual BCI for the visually impaired, which requires immediate reactions. Existing BCI systems are also not reliable enough to be deployed in accuracy-critical applications; the high-reliability 6G provides ameliorates this. Wireless BCI is more comfortable to use than the wired BCI thanks to the flexibility of use- users can move while using them [259]. They also have simpler connections. However, vibration and interferences from moving around are challenges that have to be overcome in wireless BCI [260].

### 6.10. Digital Replica

Digital replica, also referred to as digital twin [261] and physical twin [262], give a virtual duplicate of people, objects, places using sensors, AI, and communication technologies [8]. This has been adapted to mimic systems. It has been proposed to enable virtualization of a cyber-physical system, where safety and security analysis can be carried out without disrupting the live feed [261]. It supports an adaptive wireless protocol and provides hardware support to drop packets [263]. Users will be able to interact with digital replica through holographic display and VR devices. This paper proposes applying digital replica in tourism to have a remote experience of their desired tourist destinations. Other desirable applications are surgery and education; surgeons will create digital replicas of organs and perform surgery simulations. This is also good for teaching young surgeons. Digital twin systems have also been proposed to enhance business performance [264]. AI has been proposed to form a self-sustaining system with the digital replica [8]. Quantum optic communication has also been proposed as a key enabling technology for the actualization of the digital replica [26]. Digital replica is one of the new applications not in the context of 5G, proposed to be enabled by 6G; further research is required in this field.

The applications mentioned above, the 5G limitations, and how 6G wireless networks will enable them are highlighted in Table 17.

## 7. 6G Use Cases in Industries

It was shown in the preceding Section 6 the different applications such as holographic communication, tactile internet, multisensory XR applications, connected robotic and autonomous systems, and more. These applications open up new spheres that contribute to the actualization of new cases. Several use cases have been presented by researchers in the past, such as in manufacturing and health [10]. This paper broaches discussions on some use cases in Agriculture, Education, Media and Entertainment, Tourism, Transport and Logistics which have no significant focus. With 6G promising very high data rates, ultra-low latency, high reliability, and more, these use cases will become a reality. In this section, we delineate the proposed use cases.

### 7.1. Use Cases in Agriculture

It has been projected that, by the year 2050, current agricultural production must increase by 60–70% [274] to cater to the needs of the entire population; to achieve this, the pervasive use of high precision wireless technology will play a huge role [274]. Some use cases are autonomous vehicles, AR for training purposes, sensors for tracking variables on the farm, and data. Another application of precision agriculture, as suggested by [275], is automated irrigation control. Precision agriculture, also known as smart farming, will facilitate these by utilizing wireless sensor networks to monitor the farm variables and make intelligent controls. The stages of precision agriculture identified in [274] are: (i) data collection, (ii) diagnosis, (iii) data analysis, (iv) precision field operation and evaluation. This presents an opportunity to carry out farm operations more efficiently. The infusion of AI in precision energy has been proposed to improve efficiency on farms.

The application of IoT in the agricultural supply chain was also presented in [276]. Some other use cases of precision agriculture have been developed over the years [277,278]. A challenge identified with precision agriculture using wireless technology is the topology, making wireless signals susceptible to attenuation. This challenge can be eliminated with the use of IRS proposed in 6G networks. This allows deployment in non-line-of-sight environments, and it is more efficient than using relays. There are also challenges with routing protocols and energy-efficient devices. The authors in [274] reviewed several protocols and identified Zigbee and LoRa wireless protocols as ideal for precision agriculture owing to their low energy requirements. Privacy and security are also challenges identified with precision agriculture [279]. The high data rate and reliability and ultra-low latency by 6G networks will allow the optimized actualization of a fully automated farm system, which results in a more efficient process, and more output. 6G is also propitious in providing a more secure network through blockchain and other security enabling technologies treated in this paper.

### 7.2. Use Cases in Education

As a result of the COVID-19 pandemic, many schools have been forced to adopt an online training mode. There has also been growth in enrollment in Massive Open Online Courses (MOOC) in recent years, with an estimated global enrollment of 110 million in 2019. According to a survey to understand the perspective of teachers in a sample population of AR, it was shown that learning online can be enhanced by utilizing AR in training [280]. The survey also showed that AR had not been widely adopted in educational institutions, and most have had contact with it outside educational institutions. This presents a dearth in knowledge acquisition through AR.

The use of holographs and XR will help to give students a better experience while learning. A good example is the training of surgeons, in which XR presents a good platform for training. We propose the application of 6G in providing remote access to surgeons in the theatre room. This widens the reach of consultants in teaching hospitals to reach more students remotely and provide professional support to students and doctors during surgery. Additionally, the UAV provides mobile ambulance access to the network, thereby allowing communication during emergencies. To actualize this, the Doppler effect from moving the UAV and ambulance will need to be tackled. This use case will benefit from the concept of high mobility hotspot introduced in [250]. 6G networks have been shown to facilitate multi-sensory XR, and holographic communication will also optimize this experience. There is the challenge of a part of the population not being able to access online courses as a consequence of the unavailability of the internet. The ubiquitous feature of 6G will also ameliorate this challenge.

### 7.3. Use Cases in Media and Entertainment

5G is expected to double annual media revenues in the next ten years to $420 billion in 2028 [281], and gaming was posited to be at the forefront of 5G innovation. Cloud-based gaming, Over-the-Top TV, AR, and VR open up new media applications. The challenge with AR and VR applications is that they require very low latency to make delays imperceptible by human users. The ultra-low latency of 6G would circumvent these delays. This paper proposes tactile internet, XR video games, cloud gaming, holographic advertising as some of the trends in 6G that will facilitate growth in media and entertainment. This not only opens up new cases in media and entertainment, but it also provides succor to those with medical needs [282]. The perceptual experience of haptics and implants will give a new sensation different and better than the one currently obtainable. BCI will also provide the visually impaired opportunity to experience fully immersive gaming. VR has been utilized to support the mental health of health care professionals during the COVID-19 pandemic. Advertising will have a wider reach through the 6G ubiquitous connectivity feature. There is, however, a challenge of producing energy-efficient devices that will support these applications.

### 7.4. Use Cases in Tourism

Many hotels have widely adopted hotel automation based on IoT. AR use cases are also providential in improving challenges associated with accommodation, transportation, and catering solutions. A recommender system was proposed in [283] to aid tourists’ decision-making, and the authors identified a need for high bandwidth wireless network infrastructure for the system to work optimally. Smart tourism also presents innovative solutions such as self-guided tours, tourist traffic control, remote monitoring, resource management [284]. This is achievable through the infusion of IoT, cloud computing, and AI [285]. The authors in [286] presented IoT in personalized hotel rooms, voice-based interaction, inventory management, location-based information, and body sensors. Tourism was one of the hardest-hit industries because of the COVID-19 pandemic [287], and virtual reality was one of the solutions identified to help the industry lessen the effects of the pandemic [288].

Some tourism companies have already adopted this in virtual tours, booking interfaces, and other travel experiences. Tourism, education, marketing are other areas where VR and AR can be adopted [289]. Digital replica, one of the driving trends in 6G, provides tourism with a more interactive experience [290]. For smart tourism to be widely adopted, the trust of the public is desirable. An IoT-based solution that is secured was proposed in [291], and the security features 6G will bring reinforces this and make the user experience much better. This paper proposes that there will be a virtual remote experience of tourist centers through fully immersive XR and holographic communication. This will be facilitated by future wireless networks B5G.

### 7.5. Uses Cases in Transportation and Logistics

Smart Logistics is one of the driving trends towards the actualization of Smart Cities, and several applications have been identified [292]. It is propitious to providing a better logistic architecture for business [293]. IoT is one of the backbones of smart logistics [292,294]. Wireless sensor networks also provide an opportunity to carry out feasibility analysis in logistics [295], inventory management against spoilage in perishable foods warehousing [296]. RIS will be widely used in the warehouse to optimize communication between IoT devices. This makes full automation and communication seamless. There is also an opportunity to reduce fatigue and accidents in the warehouse by using fully autonomous forklifts. Enhanced communication and repair through VR and AR is another viable opportunity to be facilitated by 5G, which reduces downtime in the warehouse. Remote tracking will also be more comfortable with UAVs and CubeSats. This makes the utopian idea of a fully connected world become a reality. Thankfully, this is happening in our time!

## 8. Challenges in 6G: Standardization, Design, and Deployment

Despite the benefits 6G promises, some challenges could hamper the actualization of these networks. This paper examines the challenges of enabling technologies for the 6G networks in detail and proposes creative solutions to circumvent these challenges. It also clarifies the psychological and health issues associated with network densification and indoor wireless communication that 6G will be built. This paper categorizes the challenges based on the enabling technologies stated in Section 5. It also proposes potential solutions for these challenges. Table 18 presents challenges identified by researchers and proposed solutions, which have not been earlier captured in the challenges with the enabling technologies.

### 8.1. Signal Processing Challenges

Some signal processing challenges that currently stand in the way of the full actualization of 6G have been identified in the paper. The challenges are delineated in this section, considering challenges and probable solutions identified by researchers. The challenges can be classified into Channel Estimation, Hardware complexity, and Precoding. Channel Estimation challenges associated with RIS systems have been treated in this paper, and the issue of hardware complexity has been raised severally. The paper focuses on precoding in this section.

#### Terahertz Precoding

Precoding is a useful technique in solving the path-loss issue in THz communication [202] without increasing the transmit power. Pencil beams, which are supported by very large-scale array antennas, help to actualize this. Precoding is channel adaptive, and it processes transmitted signals based on the available channel information at the transmitter [202,297]. However, there are challenges with precoding. The authors [31] have identified high power consumption as a challenge. Other challenges posited are the beam split effect and path-loss as a result of the increasing distance. Hardware impairments such as the non-linearity of power amplifiers limit THz precoding performance [12].

### 8.2. Social, Psychological, Health, and Commercialization Challenges

**Figure 8:**
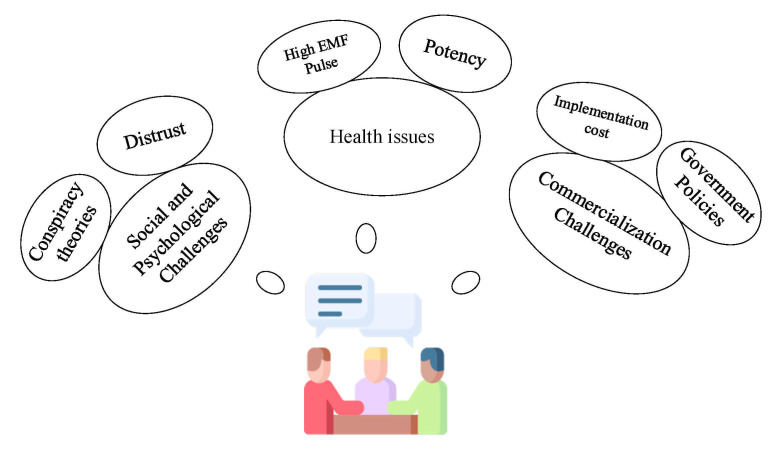
Non-wireless technology-related challenges for 6G.

Social factors can make or mar the adoption of technology, and 6G wireless communication is no exception. As humans, our psychology and environment guide many of our actions. There was a hot debate that accompanied 5G implementation. It was believed in some quarters that the high-frequency spectrum is insidious to health. Some conspiracy theorists went as far as linking the 5G network to the proliferation of the COVID-19 pandemic. In this section, we critically examine the social, psychological, and health concerns. Furthermore, we examine some impending challenges that may sprout up during 6G commercialization, and suggestions were made to tackle these challenges. Figure 8 gives a pictorial view of some of these impending issues.

#### 8.2.1. Social, Psychological, and Health Challenges

Considering the cost of implementing future wireless networks, it is important to convince governments and agencies that it is expedient to implement it. Despite the benefits technologies driven by 6G networks present, there have been concerns on the health risks of 5G and 6G, especially considering network densification and transmitting at a higher frequency spectrum [12,48]. Although transmitting at mmWave, Terahertz, and Visible light are at a higher frequency as against what is currently obtainable, they belong to the non-ionizing range. Although there have been concerns on the dangers of 5G [298] and scientists, have bemoaned and petitioned the WHO about the hazards of 5G, not just with the potency. Still, with the pulse, the world would be exposed to [299], the effects of Electromagnetic Fields (EMF) have been studied [300], and no adverse health effects have been established.

The International Commission on Non-Ionizing Radiation Protection (ICNIRP) has recently updated their exposure guideline to abide by mobile operators and telecommunication companies. The new guideline covers higher frequency bands envisaged in 5G networks and beyond [301]. It is crucial to ensure that these guidelines are adhered to. More research is conducted to ascertain the effects of higher frequency on humans and other organisms in the ecosystem and how to assuage the effects. When these issues are addressed, the social and psychological challenges expected in 5G and beyond will be reduced considerably and subsequently expunged.

#### 8.2.2. Commercialization Challenges

6G commercialization challenges were keenly discussed at the 6Genesis Flagship Program (6GFP) launched in Finland in May 2018. The 6GFP consortium comprises academia, research centers, institutes, industrial and business partners, and coordinated by the University of Oulu in Finland. The program is the first to focus on 6G research globally [302]. The Director of the 6G flagship, Matti Latva-aho, noted that 6G would be deployed around 2030 to meet the expectations not provided by 5G, alongside the new ones fusing AI-inspired applications in every field of society with ubiquitous wireless connectivity. The estimated cost of the 6G flagship will be around EUR 250 million over eight years.

There has been limited literature on the potential commercialization challenges of 6G. Although the deployment of 6G is expected to commence in 2030 [26], it is prudent to foresee the challenges associated with commercialization to prevent or circumvent these challenges. 6G networks are proposed to facilitate ubiquitous connectivity, even in rural communities. Rural areas have the challenge of lack of revenue per square mile to accommodate the enormous cost of 6G deployment. One question posed at the first 6G summit by Tarik Taleb is if autonomous companies would be willing to pay for the cost of connectivity to have access to this technology. Will smart factories need operators for their networks? Will the training costs to adapt to the new networks be worth the money? These are some of the questions that need to be answered from a business perspective. 5G deployment is posed with the challenge of cost, which is envisaged in 6G deployment.

The use of UAVs and advanced radio techniques, exploitation of solar-powered energy-efficient devices, reusability of network components and functions, and deployment of commodity hardware were identified as some of the pillars to actualize connectivity in rural areas [89]. However, this was based on the premise that internet connectivity will be a primary need that the government or government-based entities may provide, but not private entities. This presents an uncertainty on who will bear the cost of deployment. Interference of government policies was witnessed in the 5G deployment. It is not yet clear enough if 6G networks will be fully supported by the infrastructure of pre-existing generations, especially 5G, or there will be a total overhaul of infrastructure. Nevertheless, it is essential to note that the existing tower infrastructure is unsustainable, and the complete actualization of 5G and subsequently 6G will present a more sustainable business model.

Although the mmWave was presented as the enabling spectrum for 5G, early deployment of 5G has been on the sub-6GHz spectrum. Early 6G deployment may not be deployed on THz immediately at the point of deployment. The early adoption of sub-6GHz in 6G was suggested in [250]. It is, therefore, pertinent to maximize the sub-6GHz spectrum. The adoption of a wireless generation is driven by the services provided and not just the enabling technologies. Although some use cases of 6G have been proposed in this paper, the onus is on the users to adopt these services. If users are reluctant to adopt these use cases, though highly unlikely, it presents a dent in the expected fast adoption of 6G. In all, there is a need for investment in 6G research and its subsequent deployment.

## 9. 6G Sustainability and Business Model

In this section, we discuss 6G sustainability and business model. First, we present a brief discussion in Section 9.1 on the multi-faceted communication capabilities of 6G that will contribute significantly to global sustainability and offering massive support for various services to promote healthy and economic stability. Next, we describe how 6G will bring about a dramatic change in the business arena in Section 9.2.

### 9.1. 6G Sustainability

The 5G networks have drastically resolved several social issues inherent in 4G wireless networks. For example, environmental protection and education have received remarkable improvements in the 5G era. However, the problems of connectivity and urbanization remain. No doubt, 6G will usher in a great sigh of relief. User-experienced-based hyperdata connectivity will be ubiquitous and regional barriers will be broken to achieve a truly borderless society [47,302]. The multi-faceted communication capabilities of 6G will contribute immensely to global sustainability and offer massive support for various services at the application layer [303]. This will, in turn, accelerates smart farming, access to financial services, and facilitates online healthcare delivery. In terms of energy conservation, 6G will aid electronic circuit miniaturization and empower extra large-scale integration of components to reduce carbon footprint. However, achieving the Sustainable Development Goals (SDGs); data security and inclusiveness, will come at a price. All parties involved; government agencies, hospitals, and society, must meet strict privacy rules to promote the integrity of the system. 6G will provide digital infrastructure to address the needs of humans and society.

6G will also enable context-aware environment sensing and indoor localization to aid online monitoring and support the massive uplifting of the SDGs ecosystem. A summary of the set of KPIs for all 17 SDGs, including probable use cases, is presented in [303]. To provide viable solutions to the vast sustainability problems, the 6G research community needs to closely examine the open ecosystem-based value configuration and decentralized poly-nodal power configuration [304,305]. 6G will be the foci point between nanotechnology, biotechnology, cognitive science, and ICT. Ultimately, this will increase societal requirements; resilience, sustainability, transparency, and inclusivity, leading to a complex societal mix [306,307]. Additionally, 6G will provide an atmosphere of productivity and rapid economic growth in rural and urban geographies towards achieving the UN sustainability goals, especially goals 9 (Industry, Innovation, and Infrastructure), 11 (Sustainable Cities and Communities), amongst others.

### 9.2. 6G Business Model

Given the recent global financial crisis, there is no denying the fact that the existing corporate business models had a significant impact on the sustainability of the global economy and society [308,309,310]. This is a wake-up call for all companies to regulate their operations geared towards achieving the UN SDGs [303]. The business scenarios for 6G in the next ten years are envisioned as highly prosperous [302]. Blockchain-empowered decentralized resource configuration techniques have been proposed to support several verticals and business platforms in the prospective 6G wireless network [303]. The probable market trends and uncertainties are elaborated and three scenarios identified by Ziegler and Yrjola [311]. These include user experience, sustainability, and business. Scalability and sustainability are proposed to be critical drivers for future 6G business models [312,313].

**Figure 9:**
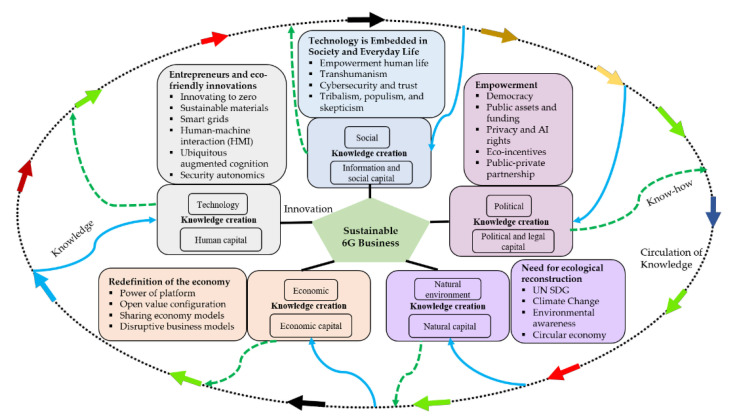
Useful choices for developing the preferred sustainable 6G business futures.

6G will bring about a dramatic change in the business arena. The future of business will be characterized by a seamless and automated collection of market data from humans and the dynamic business environment. 6G will provide a user-friendly platform for intelligent analysis of big data for high-end products and specialized services. These products and services would be designed to be highly sustainable and well-tailored to address the peculiar needs of the consumers in rural and urban geographies. Additionally, 6G will aid crowdsourcing and enhance cutting-edge distribution platforms to support sharing sustainable business models to accelerate the equitable distribution of resources. Recently, the choices for developing the first-class sustainable 6G business future for all the participants in the future 6G business were recapitulated by [307]. These useful choices for creating the favorite sustainable 6G business futures have been designed using the quintuple helix model shown in Figure 9. Sustainable 6G business is key to knowledge creation to drive human empowerment, technology acquisition and transfer, economic stability, entrepreneurship and innovation, natural environment conservation, ecological reconstruction, and democratization of government institutions.

## 10. Recent Trends, Future Research Directions, and Lessons Learned

This section considers some recent trends in 6G applications, such as Holographic communication, space communication, and more. We have also identified areas where further research is needed. We have considered some research propositions by several researchers hoping this will stimulate research and facilitate the progress, development, and rapid implementation of 6G wireless communication. We conclude the paper with a summary of key take-away lessons for further research and development in 6G wireless communication.

### 10.1. Recent Trends

China launched the first 6G satellite to space in November 2020. The satellite uses high-frequency terahertz waves for data transmission. Although the launch is just a trial, and it is not sure if it will be in the final 6G standard, this has ushered in the 3D architecture of terrestrial, space, and underwater communication, kick-starting transmission on space. There is an ongoing development of 6D Holographic optical field technology. This will be enabled by the exigent specifications of higher data rate and low latency to be facilitated by 6G. This technology presents a new and desirable user experience in media and entertainment. Security has been identified as a challenge in the adoption of the Internet of Bio-Nano Things. The authors in [249] proposed a biocyber interface-based privacy scheme. The privacy scheme worked on top of the biocyber interface in the IoBNT model and showed promising results with minimal side effects. Research in autonomous driving is progressing, and driverless buses and taxis are expected on Britain’s roads as of 2021 [314]. As explained earlier in this paper, the psychological trust of the population is desirable for the seamless adoption of this technology. Considering Terahertz transmission, spatial modulation [315] has been proposed to minimize the hardware impairments [316]. The development of Wireless Network on Chip (WNoC) enabled by miniaturized transceivers and antenna is also gaining traction [189]. We believe these trends and developments draw us closer to the realization of 6G wireless communication systems.

### 10.2. Future Research Directions

Pervasive AI is crucial in actualizing intelligent future wireless networks. However, there is a challenge of having energy-efficient hardware at the mobile unit, allowing users to experience high-speed applications such as extended reality, fully autonomous systems, and more. Therefore, further research is needed to produce hardware that will be compatible with 6G. These devices are desired to be energy efficient. This keeps in play the vision to have longer battery life. Security and trust of wireless networks is also an issue which 6G is expected to combat. There is a need for more research on quantum communication. As stated earlier in this paper, technologies such as Quantum communication, Internet of Bio-Nano Things might not be mature enough to enable 6G. Still, they will be key future wireless networks. Quantum communication not only improves security but also improves computational efficiency. Space communication has been explored in this paper. If this is incorporated with terrestrial and underwater communication, the vision of a true ubiquitous communication network will be achieved. Saeed et al. [182] envisage the CubeSats to facilitate future wireless communication in space thanks to their low cost and low orbital altitude. However, we have identified challenges in the channel model which need urgent research focus. Future research prospects in software-defined networking, Internet of Space Things, and Machine Learning resource allocation for CubeSats have been identified. Table 19 considers these open research issues on underwater communication, security, channel estimation, and more detail.

Saad et al. [47] opined that there would be a shift from smart phones-base station paradigm to smart surface–human embedded implants. They also proposed that performance analysis and optimization will require operating in the 3D space and that it requires a radio-centric design system that incorporates the 3CLS and AI. It is important to have more research on these technologies to hasten the rate of development. In addition to the future research directions identified in this paper, we have also summarized research trends broached by different researchers in Table 20. Furthermore, future projections were earlier identified in Table 4 of this paper.

### 10.3. Lessons Learned

In this section, we have identified and summarized lessons learned from this research work. We believe these lessons are crucial and will guide researchers on progressing 6G research.

***Lesson 1: Comparing the Key Technologies that will facilitate the actualization of 6G.***

It has been established that Terahertz communication and VLC will give a higher data rate than the one obtainable in 5G networks. Cell-free massive MIMO presents a new concept that eliminates cells in communication and improves user experience. UAVs and CubeSats, which form the Internet of Space Things introduced in [37], and underwater communication will play a huge role in achieving ubiquitous connectivity. Challenges with each technology have also been identified in this article. It has also been established that quantum communication and blockchain will provide secure future wireless networks. Ambient backscatter communication will also play a huge role in energy and spectral efficiency. Artificial Intelligence will be pervasively adopted to enable intelligent networks, making them self-efficient. Deep Learning has been proposed as an alternative to existing algorithms for modeling channel information. However, despite the high processing power that has expanded AI development in recent times, data are still cumbersome. We propose Big Data to play a crucial role in managing the large amount of data associated with wireless networks.

***Lesson 2: 5G is limited, and 6G is proposed to provide requirements that will be compatible with emerging applications and services.***

Current 5G systems do not have the bandwidth to support a fully immersive XR experience and high definition holographic communication. Applications such as Tactile Internet, Wireless Brain–Computer Interface also require high reliability and ultra-low latency, which 5G cannot provide. Additionally, needed is a very high data rate in the order of 1Tbps with a latency <1 ms, making delay imperceptible to humans. These will be provided by 6G. Achieving this enables new use cases in agriculture, education, media and entertainment, tourism, and logistics. Additionally, it has been established that the sub-6GHz band is crowded. There are research interests in TeraHertz spectrum communication. This will provide a new spectrum and enable higher data rates, which are desirable in 6G. Optical Wireless Technology such as Visible Light Communication and Free Space Optics also need to be explored.

***Lesson 3: Compatibility at the hardware level is still a major challenge as many UE are currently not 6G compatible.***

Artificial Intelligence is proposed to enable wireless networks to be intelligent entities. However, this casts a toll on the energy requirement at the mobile unit, as high processing power is required to process a large amount of data and the AI algorithms. Additionally, there is a need for new chips for TeraHertz communication. Wireless Network on Chip (WNoC) has been introduced in [189], but further research needs to be done to check its practicability. There is a need to have energy-efficient devices that support the driving trends in 6G. There is also a need for new protocols or updates of existing protocols to enable communication in 6G networks. In all, the hardware and protocols need to be cost-efficient to aid easy commercialization.

***Lesson 4: Will there be a complete overhaul of the backhaul of present wireless networks, or will there be new Infrastructure? Not certain.***

It is not yet clear if there will be a complete overhaul of existing wireless networks with a new architecture for 6G. However, there will undoubtedly be a need for more infrastructure, especially RIS, UAVs, CubeSats, which are yet to be widely adopted. Massive MIMO previously deployed in 5G will be further explored to incorporate a cell-free massive communication network. A 3D network architecture that combines satellite, UAV, and terrestrial networks is proposed to facilitate ubiquitous communication. This will bring about space and terrestrial communication. It is also crucial to incorporate underwater communication. It is worthy to note at this juncture that it will be desirable to incorporate the existing architecture, especially those of the recently commercialized 5G. This enables a network that is not just performance efficient but cost-efficient.

***Lesson 5: 3CS (Communication, Computing, Control).***

6G has been proposed to be a multi-purpose system that combines communication, computing, control, and localization [47]. The authors in [6,331] went further to include caching. The combination of communication, computing, and control is an aberration from what is obtainable in existing generations where the focus was solely on wireless communication. However, challenges such as enabling intelligent control in stringent applications such as autonomous vehicles, multi-modal fusion for 3D image reconstructing [47], and lack of prior models exist. Quantum communication is proposed to play a key role in improving the speed and performance of computational processes. The actualization of these will facilitate CRAS, XR, and more applications

***Lesson 6: Haptics will be more prominent.***

Haptics present a form of communication using touch. Haptic devices have a unique bi-directionality feature that allows them to communicate with the brain, i.e., bi-directional communication with the brain. Teletouch facilitated by Haptics technology will remake the internet into allowing users to move distant objects and experiencing instant tactile feedback. Upon the wide adoption of haptics, it has been proposed to be the end of the smartphone era [47], a new era of wearables and haptics will be ushered in. Applications such as multisensory extended reality, BCI will also fuel this transition. However, for these technologies to be widely adopted, the psychological concerns of the users will need to be resolved.

***Lesson 7: Industry 4.0 will be facilitated by CRAS, smart logistics, precision agriculture, smart cities.***

There will be a pervasive application of AI and automated control systems. This will open up applications such as CRAS that will improve industrial processes and products effectively. Industry 5.0 will be more user-centric with the personalized manufacturing process. Holographic communication, Multisensory XR, Digital Twin, and more will facilitate the actualization of smart cities. This will also bring about an upgrade in the media and entertainment industry. There will be a better user experience in online gaming through extended reality, tactile devices, and more. New use cases in smart logistics, precision agriculture, and more have been introduced in this paper, and these are expected to drive massive industrialization. These applications and use cases make the actualization of 6G desirable.

***Lesson 8: Security, a desirable feature of 6G, will be provided by blockchain and quantum communication.***

There are research interests in quantum cryptography and quantum machine learning. This is expected to make future wireless networks secured. However, quantum communication research has not received as much attention as other enabling technologies; thus, casting doubts on its maturity in 6G deployment. Therefore, there is a need for more research focus on quantum communication. Blockchain is another technology that will improve the trust and security of future wireless networks. This is a distributed ledger technology with decentralized control. Artificial Intelligence will also play a role in detecting threats in wireless networks, thus creating a secure and self-sustaining network.

***Lesson 9: Need for a new networking protocol.***

There is also a need for new networking protocols or an upgrade to the existing ones to be compatible with the new technologies such as the THz frequency spectrum, which are enablers of 6G. As stated earlier in this paper, communicating at a higher frequency spectrum is prone to high path loss. Therefore, there is a need for hardware to improve the transmission range of THz communication. There is also a need for a robust system and algorithm for handover management in UAV/CubeSat communication. It is as well crucial that these hardware architectures are low-cost and energy-efficient. Additionally, as stated earlier, compatibility with existing infrastructure is desirable.

***Lesson 10: Multiplexing methods.***

Multiplexing techniques such as Orthogonal Frequency Division Multiplexing (OFDM), Generalized Frequency Division Multiplexing (GFDM), Filter Bank Multicarrier (FBMC), Spatial Multiplexing, and more, are proposed in 5G. Further research is needed to ascertain how these multiplexing methods will be crucial in actualizing the desirable 6G networks. Spectrum and infrastructure sharing is also desirable to maximize capabilities, and CR and Blockchain are some of the technologies proposed. Further research is needed to achieve this. Generally, there is a need for new protocols and standards for 6G to enable standardization and compatibility. Additionally, software-defined networking (SDN) needs to be explored to resolve protocol incompatibility.

***Lesson 11: Need to provide pragmatic solutions to the vast sustainability problems by adopting innovative and sustainable business models.***

Sustainable business is key to knowledge creation to drive human empowerment, technology acquisition and transfer, economic stability, entrepreneurship and innovation, ecological reconstruction, and democratization of government institutions. However, most current business models are not sustainable. There is a need for adaptive and innovative business models to regulate the market forces to achieve global economic stability. Ultimately, such business models will accelerate productivity and rapid economic growth in rural environments towards achieving the UN sustainability goals.

## 11. Conclusions

This paper attempted to discuss how the 6G wireless network will enable cutting-edge applications, which the existing 5G has not seamlessly facilitated. The enabling technologies for 6G wireless networks are discussed in detail and contrasted with the existing 5G technology. The challenges which pose as barriers to the actualization of 6G wireless communication are emphasized. The accompanying social, psychological, health, and commercialization challenges are discussed. New use cases of 6G in agriculture, education, media and entertainment, tourism, transport, and logistics, are broached. Channel estimation, security, underwater communication, and more require further research to guarantee the envisioned ubiquitous connectivity in the 6G era and beyond. The multi-faceted 6G wireless communication capabilities in accelerating the United Nations Sustainable Development Goals to drastically alleviate poverty, promote an equitable and fair distribution of scarce resources, improve healthcare delivery, and maintain global economic stability are discussed extensively. Finally, we have given a concise but more explicit vision of what 6G will be, provide an up-to-date review of the emerging trends, assess the potential challenges envisioned in 6G wireless network deployment, and stimulate research initiatives to tackle these challenges for societal advancement.

## Figures and Tables

**Table 1:** Limitations of some related surveys and our contributions.

Ref.	Focus and Coverage	Limitations	This Paper’s Contributions
[45]	Considers the vision, applications, research activities, challenges, and potential solutions.	▪ Applications were limited to five. ▪ Challenges with each enabling technology were omitted.	▪ More applications such as teleoperated driving, IoBNT, Digital replica, and more are treated in this paper. ▪ Provides a holistic review of the challenges of each technology. ▪ Additionally, it analyses enabling technologies not treated, such as RIS, CubeSats, ABCS, and more. ▪ Examines the most recent research trends and highlights future directions and lessons learned.
[9]	Vision and potential techniques. The study presents some technology-driven challenges such as power supply, security, hardware design, and probable solutions.	▪ The space-air-ground integrated network was proposed, but the supporting technologies of UAV/CubeSats were not explained. ▪ Applications not clearly outlined.	▪ An extensive analysis of UAV/Cubesats and how these will facilitate 6G requirements are presented. ▪ 6G applications are clearly outlined, and a comparative analysis of why the existing 5G is limited is shown. ▪ Enabling technologies such as Cell-Free massive MIMO, ABCS, quantum communication not presented in the paper were delineated in this paper.
[33]	The authors examine the vision, requirements, and Services.	▪ The enabling technologies are not outlined. ▪ Future research directions not presented.	▪ Vision, Requirements, Applications, and Enabling Technologies are discussed robustly. ▪ Open Research Issues and Future Research Directions outlined.
[41]	This work presents an extension to the existing vision of 5G and shows speculatively how the 5G vision and technologies can be enhanced to drive the anticipated 6G.	▪ Although use cases were discussed, the driving applications were omitted. ▪ Open Research Issues and Future Research Directions not clearly outlined.	▪ Driving trends and applications extensively discussed. Challenge with each enabling technology clearly outlined. ▪ Open Research Issues and Future Research Directions are clearly outlined.
[40]	6G vision, key features, potential applications, enabling technologies. Emphasizes critical features such as security, secrecy, and privacy to make 6G truly human-centric. The system framework, key technologies, and challenges are outlined to support the 6G vision.	▪ Challenges with enabling technologies are not clearly outlined. ▪ Applications such as IoBNT, digital replica not considered.	▪ Enabling Technologies such as Blockchain, ABCS discussed. ▪ Challenges with enabling technologies are clearly outlined. ▪ Applications such as IoBNT, digital replica, and wireless BCI are discussed. ▪ Research activities updated to include the most recent research activities.
[46]	The work presents topics in human-machine interface, multi-sensory data fusion, ubiquitous computing, and precision sensing. The authors added key disruptive technologies that include new architecture, new security, and a new spectrum.	▪ Enabling Technologies limited to six and did not include technologies such as blockchain, ABCS. ▪ Applications not clearly outlined.	▪ Enabling Technologies and challenges are discussed extensively. ▪ Additionally, the existing and probable solutions to some of these challenges are highlighted ▪ 6G Applications are clearly outlined.
[47]	The authors present a vision on 6G, considering the applications, service classes, essential requirements, and trends. The enabling technologies and open research problems were highlighted.	▪ Enabling Technologies discussed, but the challenges were not clearly outlined. ▪ Additionally, non-technical challenges such as commercialization and psychological challenges are not discussed.	▪ Arobust discussion on Blockchain as an emerging technology is reported. ▪ Technical challenges associated with the enabling technologies and non-technical challenges such as commercialization are discussed.
[48]	This survey focuses on 6G applications, requirements, challenges, and critical areas of research focus. The survey also covers key technologies such as THz, blockchain, AI, and optical wireless communication (OWC).	▪ The challenges with the enabling technologies proposed are not discussed. ▪ Use cases not discussed.	▪ Discusses the challenges with enabling technologies. ▪ Presents use cases in education, media, entertainment, and more. ▪ Examines the proposed applications, the requirements, and why existing 5G cannot meet the requirements.
[49]	Presents the enabling technologies, including the holographic radio characteristics and targeted application scenarios. Additionally, considers non-technical challenges such as industry barrier and consumer habits.	▪ Applications not clearly outlined. ▪ Open research issues and future research directions not discussed.	▪ More enabling technologies are discussed. ▪ Technical and non-technical challenges are presented. ▪ 6G applications are clearly outlined. ▪ Open research issues, future research directions, and lessons learned are outlined.

**Table 2:** Comparing the different generations of wireless communication from 1G to 6G.

Features	1G	2G	3G	4G	5G	6G
Technology	AMPS [59], IMTS, PTT	GSM [60], GPRS, CDMA [61], EGDE	WCDMA [62], UMTS, TD-SDMA, CDMA2000 [63], WiMAX [64]	LTE [65], MIMO [66]	Massive MIMO, network densification, millimeter- wave transmission	RIS [67], Cell-free Massive MIMO [68], Terahertz spectrum [69], AI [24]
Data-rate range	>3 kbps	10 kbps–200 kps	300 kbps–30 Mbps	100 Mps–1000 Mbps	1–30 Gbps	100 Gbps–1 Tbs
Latency	>1000 ms	300 ms–1000 ms	100 ms–500 ms	20 ms–100 ms	1 ms–10 ms	<1 ms
Multiple Access/ Multiplexing schemes	FDMA [70]	TDMA, CDMA	CDMA [71]	OFDMA [72]	OFDM, GFDM [73] FBMC [74], Adaptive Time-Frequency. Multiplexing [75]	OMA [76], NOMA [76], OAM [77], Spatial Multiplexing [78]
**Applications**	Calls, Fax	Encrypted and data services	Faster Data, Video calling, remote supervision systems	HD Television content, Online Gaming	Internet of Things [5], Virtual reality, Immersive gaming	Autonomous systems, tactile devices, Internet of Everything, BCI, Telemedicine

This table compares the different generations of wireless communication from 1G to 6G with respect to the supporting technologies, data features, and enabling applications. It shows that with higher data rates and lower latency, more sophisticated applications are enabled.

**Table 3:** A comparative analysis of 5G, B5G, and 6G.

Description	5G	Beyond 5G	6G
Frequency bands	▪ Sub-6GHz ▪ mmWave for fixed access	▪ Sub-6GHz ▪ mmWave for fixed access	▪ Sub-6GHz ▪ mmWave for mobile access ▪ Exploration of higher frequency and THz bands (above 300 GHz) ▪ Non-RF (optical, VLC)
Rates requirements	20 Gb/s	100 Gb/s	1 Tb/s
Radio only delay requirements	100 ns	100 ns	10 ns
End-to-End delay(latency) requirements	5 ms	1 ms	<1 ms
Processing delay	100 ns	50 ns	10 ns
Device types	▪ Sensors ▪ Smartphones ▪ Drones	▪ Sensors ▪ Smartphones ▪ Drones ▪ XR equipment	▪ Sensors and DLT ▪ CRAS ▪ XR and BCI ▪ Smart implants
Architecture	▪ Dense sub-6 GHz small base stations with umbrella macro stations. ▪ mmWave small cells of about 100 m (about fixed access).	▪ Denser sub-6 GHz small cells with umbrella macro base stations. ▪ <100 m tiny and dense mmWave cells.	▪ Cell-free smart surfaces at high frequency supported by mmWave tiny cells for mobile and free access. ▪ Temporary hotspots are served by drone-carrier base stations or tethered balloons. ▪ Trials of tiny THz cells.
Services	▪ eMBB ▪ URLLC ▪ mMTC	▪ Reliable eMBB ▪ URLLC ▪ mMTC ▪ Hybrid (URLLC + eMBB)	▪ HCS ▪ MPS ▪ MBRLLC ▪ mURLLC

This table zooms in on the comparison of 5G, Beyond 5G, and 6G, presenting a specific latency, brief description of the architecture, and services such as MBRLLC, mURLLC that have been proposed. The device types are also shown to include BCI and smart implants in 6G.

**Table 7:** 6G research in selected countries and regions.

Country	Year	Research Initiatives
Finland	2018	The consortium comprises academia, research centers, industrial partners, business units, and others. This is coordinated by the Centre for Wireless Communication, University of Oulu, Finland.
		The 6Genesis project is focused on research that embraces electronics and materials, wireless communication, computer science, and engineering. First 6G Wireless Summit was held in Levi, Lapland, Finland, in March 2019.
United States	2019	The Federal Communications Commission opened the spectrumbetween 95 GHz and 3 THz to create a new category ofExperimental licenses.
		BWA Lab working on Ultra-MIMO based Intelligent Network
		ComSenTer researching wireless communication in the THz region. Research ongoing by NYU Wireless, VIVO communication Research Institute.
China	2019	Ministry of Science and Technology in 2019 announced the establishment of two groups for 6G research: One group will consist of relevant government departments, and the other team will be made up of 37 universities, research institutes, and enterprises, which will lay out the technical side of 6G and offer advice.
		ZTE has a group on advanced technologies, researching networks beyond 5G. Huawei also commenced research at their research facility in Ottawa, Canada. They also have a research facility in Segrate, Italy, where research on microwave communication, frequency spectrum, and transmission speed is carried out.
The Netherland	2019	The Eindhoven University of Technology developed new antenna technology.
EU	2019	Networking Research beyond 5G, an EU-Japan project, is investigating the possibility of THz spectrum from 100 GHz to 450 GHz.
		Terranova project, a research group by the EU, is working toward a reliable 6G connection with 400 Gigabit per second transmission capability in the terahertz spectrum.
		6th International Telecommunication Union Workshop on networks in 2030 held in Lisbon, Portugal in January 2020.
South Korea	2019	LG partnership with the Korea Advanced Institute of Science and Technology to conduct 6G research. Samsung also kicked off 6G research in 2019.
		SK Telecom announced a collaboration with Nokia and Ericsson on 6G research in 2019.
Russia	2019	The Polytechnic Institute of Physics, Nanotechnology, and Telecommunications are researching 6G.
Germany	2019	TU Berlin introduces an Einstein Fellowship to study 5G and 6G.
Japan	2020	A committee of professionals from the private sector and university researchers to investigate the challenges of 6G. Sony, NTT Docomo, and Intel partner to carry out 6G research.
		Osaka University and Nanyang Technological University Singapore partner to develop a chip that allows an 11Gbps data rate.
Germany	2020	The German government set aside 50 billion Euros to develop future wireless technologies, including 6G, quantum computing.
United Kingdom	2020	The University of Surrey launched 6GIC in November 2020.
China	2020	In November 2020, China launched the first 6G test satellite into orbit.

This table shows the initiatives by different countries, from the flag-off in Finland in 2018 and the recently launched satellites by China in 2020. Different research initiatives by different countries are also highlighted, and some novel innovations are also stated.

**Table 9:** Types of metasurfaces being used for the design of 6G wireless networks.

Reconfigurable Intelligent Surfaces	
Structure	Patch-array based
	Metamaterial based
Tuning mechanisms	Active
	Passive-lossy
	Passive-lossless
Power source	Waveguide RIS
	Reflecting/Refracting RIS
Energy consumption	Electrical Excitation
	Thermal Excitation
	Mechanical stretching

This table classifies metasurfaces based on structure, tuning mechanisms, power source, and energy consumption.

**Table 11:** A comparison of UAVs and CubeSats.

UAV Communication	CubeSat Communication
Operates at low altitude, usually no more than a few kilometers.	Operates at high altitude.
Flexible and more comfortable to control and configure to suit the environment.	Not as flexible.
Capable of moving in 3-dimensional movements.	Limited to 2-dimensional movements.
Higher power consumption but can be improved.	Low Power Consumption, utilizing solar power generation.

Compares the features of UAVs and CubeSats. UAVs fly at a lower altitude when compared to CubeSats. UAVs can move in 3D and act as relay nodes that incorporate terrestrial and non-terrestrial communication.

**Table 12:** Comparison of mmWave, THz, VLC and FSO.

Network Characteristics	mmWave	THz	VLC	FSO
Frequency range	30–300 GHz	0.3–10 THz	400–800 THz	187–400 THz
Wireless Generation	5G	B5G	4G, 5G, B5G	4G, 5G, B5G
Data rate	1–20 Gbps	100 Gbps–1 Tbps	4–15 Gbps [196]	13 Tbps
Hardware architecture	Large scale antenna array at the BS [197]	Photonics and Electronic based hardware [31]	LEDs and Laser Diodes (LD) in Visible light range	Point-to-point lasers in the infrared range
Transmission distance	5 km	10–100 m	15 cm–197 cm [196]	4 km
Applications	Telecommunication, radio astronomy, remote sensing, automotive radars	Wireless cognition, hyper-active position location, sensing, and imaging	Indoor and Outdoor communication	Outdoor communication, Storage Area Network, Military access [195]

This table compares THz transmission with other higher frequency transmissions such as mmWave, and emerging OWT such as VLC and FSO. The hardware architecture, applications, and more are also considered.

**Table 13:** Differences between traditional backscatter and ambient backscatter communication systems.

Ambient Backscatter Communication	Traditional Backscatter Communication
Utilizes ambient RF	Requires special-purpose power infrastructure, e.g., RFID reader
Provides device-to-device(D2D) communication	Does not provide D2D communication
Energy-efficient as ambient energy is utilized	Not as energy-efficient

This Table compares and contrasts ambient backscatter communication with traditional backscatter communication. This is with respect to the mode of operation, energy efficiency, and more.

**Table 14:** Assessment of the enabling technologies and the desired requirements in 6G.

Enabling Technology	High Data Rate	Security, Privacy, Integrity	Ultra-Reliable Low Latency	Spectrum Allocation and Efficiency	Scalability	Energy Efficiency	User-Centric Services	Ubiquitous Connectivity
Pervasive AI	✓	✓		✓	✕	✓	✓	✓
IRS	✓	✓				✓	✓	✓
UAV/CubeSat			✓	✕				✓
Blockchain		✓	✓	✓	✕			
TeraHertz/OWT	✓	✓	✓					✓
Cell-Free Massive MIMO	✓		✓	✓	✓	✓	✓	✓
Quantum Communication	✓	✓						
Ambient Backscatter Communication	✓					✓		

✓—Support; ✕—Do not support Blank- Link not established. This table maps the enabling technologies treated to selected requirements such as energy efficiency, high data rate, and more. Additionally, requirements that some enabling technologies do not favor or serve more as challenges are noted. The blank spaces are requirements that have no direct link with the mapped enabling technology, e.g., ambient backscatter is more directly related to energy efficiency, and the link has not been established with latency and other fields that are conspicuously blank.

**Table 15:** Brief description of 6G applications.

Applications	Brief Descriptions
Holographic Communication	This enables human communication through holographs-3D images in thin air. To improve the experience of remote communication as we embrace a borderless workplace. Latency and high bandwidths are some of the challenges associated with Holographic Communication. 6G will solve these challenges.
Tactile Internet	Enables human-to-machine interactions and machine-to-machine interactions.
Industry 4.0 and beyond	Comprises cyber-physical systems, IoT, and cloud computing. Additionally, AI and ultra-fast wireless networks will drive the 4th industrial revolution. This enables smart cities, factories which are some of the vision for 6G.
Teleoperated Driving	Allows cars to be controlled remotely. These cars are also referred to as semi-autonomous vehicles. Semi-autonomous cars require a fast and ubiquitous wireless network with ultra-low latency.
Internet Bio-Nano Things	An interconnection of biological nano-sized objects(nanomachines). Takes application largely in healthcare. 6G is proposed to provide the perceptual requirements and ultra-low latency required by IoBNT.
Multisensory XR Applications	AR/MR/VR that incorporates perceptual experience. Supported by URLLC and eMBB and perceptual factors to be supported by 6G. An excellent candidate to provide a better gaming experience.
Blockchain and Distributed Ledger Technologies	Blockchain is postulated to provide security for 6G networks. They also require low latency, reliable connectivity, and scalability, which 6G networks will provide.
Connected Robotics and Autonomous Systems (CRAS)	CRAS is required to improve industrialization through the use of robots and autonomous systems for industrial operations. They require a high rate and reliability and low latency.
Wireless Brain–Computer Interface (BCI)	BCI enables the communication between the brain and electronic devices. This requires ultra-low latency, high reliability, and high data rate.
Digital Replica	These are also called digital twins, and they create a digital copy to replace people, places, systems, objects. This requires a very high data rate, which 6G will enable.

This table acts as a guide for the applications considered in this section. The brief descriptions are highlighted to give the reader a background knowledge of the applications, which would be expatiated as the section progresses.

**Table 16:** Comparing AR, VR, and MR.

Parameter	AR	VR	MR
**Experience**	Overlays virtual object in the user’s environment	Immerses user in a fully artificial environment	Combines AR and VR
**Required Bandwidth**	Multiples of Gbps	Multiples of Gbps [254]	Multiples of Gbps
**Required Latency**	<15 ms	<15 ms [254]	<15 ms
**Examples**	Snapchat lenses, Pokemon Go	Google Cardboard, HTC Vive, Oculus Rift	Microsoft HoloLens

**Table 17:** Comparative analysis of the proposed applications in 5G and 6G.

Applications	5G Proposal	5G Limitation	6G Facilitation
**Holographic Communication**	To provide high bandwidth, low latency, security, and low energy footprint [265].	The maximum data rate from tests is 8 Gbps [232], and 1 Gbps at a distance of 6.5 km [233], which is below 1Tbps proposed for seamless communication [39].	Higher data rate through THz/OWT and other enabling technologies highlighted in Table 14. Superfine time synchronization and ultra-low latency [266]. ABCS to improve energy efficiency and to have a low energy footprint on the environment.
**Tactile Internet**	A latency of 1ms and an ultra-reliable network are proposed to enable tactile internet [21].	Results from 5G tests show a latency of 10-16ms, which is insufficient to support tactile internet fully.	Technologies such as UAV [267], blockchain [214], OWT [48], cell-free massive MIMO [22] to facilitate ultra-reliability and ultra-low latency.
**Industry 4.0** **and beyond**	High reliability, ultra-low latency, security, and privacy [268]. Mobility, energy efficiency is also desirable [269].	Lack ubiquitous connectivity. New modes of cyberattacks [270].	To enable ultra-reliability and ultra-low latency. Blockchain and quantum communication to improve security and privacy. ABCS to improve energy efficiency.
**Teleoperated Driving**	Massive simultaneous connection and ubiquitous connectivity to enable autonomous and semi-autonomous vehicles [271]. Handover management and good signal strength [272].	Lack of ubiquitous connectivity. Limited data rate with increasing distance.	The integration of Space-Ground-Air-Water communication expands the frontiers of communication and provides ubiquitous connectivity. AI to improve handover management [42].
**Internet of Bio-Nano Things (IoBNT)**	Not extensively proposed in the context of 5G.	Not extensively proposed in the context of 5G	Some works of literature have proposed IoBNT for beyond 6G (e.g., [31]), and we believe high reliability is desirable for these applications.
**Multisensory** **XR**	Low latency, high bandwidth, low power, security [268].	Although existing 5G networks can meet the minimum required latency of 15ms [254] for AR/VR, to support a fully immersive multisensory XR, a lower latency (i.e., less than 10ms) is desirable.	New enabling technologies proposed to facilitate ultra-low latency (<1 ms), higher bandwidth, ultra-reliable, and secured wireless network. These are highlighted in Table 14.
**Blockchain** **and DLS**	High data rate (1–20 Gbps), low latency in the order of 1–10 ms, and blockchain provide improved security [216].	The highest data rate achieved is 8 Gbps and latency is still within the range of 10–16 ms.	High data rate by THz transmission and Optical Wireless Technology (OWT). Additionally, AI, cell-free massive MIMO, and even then not fully developed quantum communication would make this a reality.
**CRAS**	High reliability, ultra-low latency, and security are desirable.	Tests have shown variable data, which is not consistent. The authors in [273] proposed a “safety-relevant” network requirement of <12 ms latency and <6 ms jitter. The current latency range of 5G goes above 12 ms.	To provide high reliability and ultra-low latency. The enabling technologies to facilitate this have been discussed earlier under tactile internet.
**Wireless BCI**	Not extensively proposed	Do not have Quality of Physical Experience (QoPE) to support the emerging stringent requirements [257].	To enable QoPE [47]. Wireless approaching nano-communication and AI developing rapidly to enable edge intelligence [257]. Consequently, Brain Controlled Vehicles (BCV), emotion-driven devices, will become a reality. High reliability for the network.
**Digital Replica**	It is not extensively proposed in the context of 5G.	It is not extensively proposed in the context of 5G.	Better network features, leveraging on the data requirements which will also support holographic communication and immersive XR.

**Table 18:** 6G standardization, design, and deployment challenges and proposed solutions.

Ref.	Challenges	Proposed Solutions
[82]	The problem of global coverage. Limited system capacity. Challenge of deploying computing and AI capabilities. Hardware constraints on Edge AI. Challenges associated with actualizing 3D architecture.	Adoption of mobile satellite communication. Spectrum optimization. Collaborative intelligent computing with cloud and edge. Introduction of lightweight edge AI algorithms. New 3D core network that allows intelligent mobility management and has optimized TCP/IP protocols.
[41]	Fixed access network capabilities lagging. Interference owing to network densification. Hardware constraints.	Free Space Optical Communication and quantum communication. New mathematical tools for analysis and optimization. Need for new materials that are compatible with 6G communication.
[9]	Limited lifespan of smartphones. Security Issue. The issue with miniaturizing HF transmitters and receivers.	New mobile device architecture that allows wireless power transfer and wireless energy harvesting. Low hardware complexity was also proposed. Advancement of PHY security techniques introduced in 5G. Optoelectronic Integration.
[48]	Integrating different architecture into a single platform. Compatibility of devices using 5G technology with 6G services such as XR, 1Tbs data rate.	Nil
[95]	Achieving accurate beamforming in high mobility cases. Latency results from retraining DL models on account of the fickle nature of channel state and network topology. Non-compatibility of hardware and software non-generality. Security.	DL-based hardware and software solutions. Outdoor positioning with centimeter-level accuracy was also proposed. Prioritizing executing tasks by hierarchy, cross-layer optimization, and end-to-end DL schemes. Digitalization, Visualization, and DL. Additionally, advanced positioning Technology. Joint physical layer and cybersecurity with quantum methods.
[45]	The trade-off between High Intelligence and Privacy. Complex computation for getting the balance between Security and Spectral Efficiency. Balancing SE and EE.	An intermediary between end-user data and AI. Encryption algorithm, PHY layer security with spectral loss, and AI. Energy harvesting, IRS solutions.
[108]	Limited capacity and latency issues.	Nil.

**Table 19:** 6G related open research issues that require further investigation.

Open Research Issues	Research Directions
Channel Estimation	The use of spatial correlation [317], Low-Complexity Channel Estimation [318], Comprehensive sensing technique [319], Deep Learning-based technique [320], and more, in the context of 5G, can be adapted to resolve the Channel Estimation challenges in 6G.
Security	Quantum communication has been highlighted to improve the security and privacy of wireless networks [88]. Access control and authentication challenges in blockchain need to be resolved. Further research on molecular communication security, which utilizes biochemical signals to transmit information, is required [321].
Underwater Communication	The underwater optical communication system (UOWC) [322], similar to VLC, application of acoustic signals requires further research to resolve the limited short-range problem. The need to integrate aerial, terrestrial and underwater communication is imperative. Further, the need for cutting-edge networking protocols to accelerate this integration is not negotiable.
Energy Efficiency	There are tradeoffs with hardware complexity and energy requirements. However, sustainable and efficient energy harvesting sources can also be explored [323] to limit carbon emission [324]. 5G New radio was designed to aid energy efficiency in 5G. However, the current 5G networks consume more power than 4G networks [1]. Therefore, research is needed to reduce the softwarization cost in the existing 5G networks towards 6G.
Spectral Efficiency	6G is expected to leverage on higher spectrums: subTerahertz, Terahertz, optical frequency, and more. In 5G, Cognitive Radio (CR) improves the SE through dynamic spectrum sharing [325]. However, there exists a challenge in heterogeneous networks (HetNets) with varying nodes [326]. A blockchain-based system for network sharing is envisioned [327]. These and other resource-sharing options such as FlexRAN and LayBack need further research focus [328].
Hardware Complexity	Considering the RIS and THz for 6G, the physical layer is expected to get more complicated, and this poses severe challenges to hardware design for THz communication. The appropriate communication systems and networking protocols that aid compatibility need further research. Further research is required on the scalability of Wireless Network on Chip (WiNoC) models to sustain transmission at THz over a long distance [329].

**Table 20:** Proposed Research Directions in 6G Wireless Communication.

Ref.	Future Research Directions
[6]	VLC is expected to facilitate a higher data rate. Utilizing semantic communication to circumvent the challenges of limits predicted by Shannon’s theory.
[10]	AI to facilitate a fully user-centric network architecture. Research on unsupervised and reinforcement learning, which do not require learning to drive a fully autonomous network.
[40]	High security, secrecy, and privacy, high intelligent network. Need for better battery life.
[45]	Need for a new transceiver design that will be compatible with THz propagation. Investigation of health concerns on THz transmission. Development of 3D frequency.
[80]	Research on high-speed, low complexity, and low power consumption signal processing technology.
[81]	AI-based PHY end to end architecture. Increased spectrum resources through THz, laser, VLC communication, and spectrum sharing techniques. Secured network facilitated by Quantum communication.
[41]	A shift from the electronic era in 5G to the optical and photonics era. Actualization of the full potential of the cognitive radio.
[47]	Further research in quantum computing and communication. The need for scalable AI to facilitate low-latency, and highly reliable networks. New protocols for wireless devices compatibility.
[48]	A new transceiver architecture for THz communication. Need for effective spectrum management techniques and spectrum-sharing strategies Beamforming through massive MIMO systems.
[95]	Leveraging AI for optimized performance. Blockchain for security. Quantum communication for improved speed. Application of meta-learning, light-weight NN, capsule NN for intelligent 6G. Partnership with health and social sciences to develop environmentally friendly devices that will be acceptable.
[1]	The need for human-centric services as against machine-centric and data-centric services. Vision of a space-air-ground-sea integrated network. Research on the security of wireless networks through quantum-based, blockchain-based, AI-based methods, and more.
[330]	Need for new network topology. Expansion of network spectrum. Incorporation of the non-terrestrial networks.

## Data Availability

Data sharing is not applicable to this article.

## List of Acronyms

**Abbreviation**	**Full Meaning**
3CLS	Communications, Computing, Control, Localization and Sensing
3GPP	3rd Generation Partnership Project
5G	Fifth Generation
6G	Sixth Generation
ABCS	Ambient Backscattering Communication Systems
ANN	Artificial Neural Network
AI	Artificial Intelligence
AP	Access Points
B5G	Beyond 5G
BS	Base Stations
CRAS	Connected Robotic and Autonomous Systems
DL	Deep Learning
DLT	Distributed Ledger Technology
DNN	Deep Neural Network
eMBB	Enhanced Mobile Broadband
EE	Energy Efficiency
EH	Energy Harvesting
FSO	Free Space Optical
GUE UL	Ground User Uplink
IoBNT	Internet of Bio-Nano Things
IoE	Internet of Everything
IoT	Internet of Things
mBBL	Mobile Broadband Bandwidth Additionally, Low Latency
mBBMT	Massive Broadband Bandwidth Machine Type
mLLMT	Massive Low Latency Machine Type
mURLLC	Massive Ultra-Reliable Low Latency Communication
MIMO	Multiple Input Multiple Output
ML	Machine Learning
MR	Mixed Reality
MU	Mobile User
NN	Neural Network
NOMA	Non-Orthogonal Multiple Access
LEO	Low Earth Orbit
Li-Fi	Light Fidelity
LIS	Large Intelligent Surfaces
LTCC	Low-Temperature Co-fired Ceramic
LTE	Long Term Evolution
OAM	Orbital Angular Modulation
OWT	Optical Wireless Technology
RIS	Reconfigurable Intelligent Surfaces
SE	Spectral Efficiency
SIWPT	Simultaneous Information and Wireless Transfer
TDD	Time-Division Duplexing
U2U	UAV-to-UAV
UAV	Unmanned Aerial Vehicles
URLLC	Ultra-reliable, Ultra-low Latency Communication
VLC	Visible Light Communication
VR	Virtual Reality
WPT	Wireless Power Transfer
QoE	Quality of Experience
QoPE	Quality of Physical Experience
QoS	Quality of Service
XR	Extended Reality
